# Buffering by gene duplicates: an analysis of molecular correlates and evolutionary conservation

**DOI:** 10.1186/1471-2164-9-609

**Published:** 2008-12-16

**Authors:** Kevin Hannay, Edward M Marcotte, Christine Vogel

**Affiliations:** 1 Institute for Cellular and Molecular Biology, Center for Systems and Synthetic Biology, University of Texas at Austin, 2500 Speedway, MBB 3. 210, Austin, TX 78712, USA

## Abstract

**Background:**

One mechanism to account for robustness against gene knockouts or knockdowns is through buffering by gene duplicates, but the extent and general correlates of this process in organisms is still a matter of debate. To reveal general trends of this process, we provide a comprehensive comparison of gene essentiality, duplication and buffering by duplicates across seven bacteria (*Mycoplasma genitalium, Bacillus subtilis, Helicobacter pylori, Haemophilus influenzae, Mycobacterium tuberculosis, Pseudomonas aeruginosa, Escherichia coli*), and four eukaryotes (*Saccharomyces cerevisiae *(yeast), *Caenorhabditis elegans *(worm), *Drosophila melanogaster *(fly), *Mus musculus *(mouse)).

**Results:**

In nine of the eleven organisms, duplicates significantly increase chances of survival upon gene deletion (P-value ≤ 0.05), but only by up to 13%. Given that duplicates make up to 80% of eukaryotic genomes, the small contribution is surprising and points to dominant roles of other buffering processes, such as alternative metabolic pathways. The buffering capacity of duplicates appears to be independent of the degree of gene essentiality and tends to be higher for genes with high expression levels. For example, buffering capacity increases to 23% amongst highly expressed genes in *E. coli*. Sequence similarity and the number of duplicates per gene are weak predictors of the duplicate's buffering capacity. In a case study we show that buffering gene duplicates in yeast and worm are somewhat more similar in their functions than non-buffering duplicates and have increased transcriptional and translational activity.

**Conclusion:**

In sum, the extent of gene essentiality and buffering by duplicates is not conserved across organisms and does not correlate with the organisms' apparent complexity. This heterogeneity goes beyond what would be expected from differences in experimental approaches alone. Buffering by duplicates contributes to robustness in several organisms, but to a small extent – and the relatively large amount of buffering by duplicates observed in yeast and worm may be largely specific to these organisms. Thus, the only common factor of buffering by duplicates between different organisms may be the by-product of duplicate retention due to demands of high dosage.

## Background

Cells and organisms show a remarkable robustness against loss of one or more genes, which has triggered an ongoing discussion on the factors promoting such robustness [1,2]. One of the simplest and most obvious mechanism for buffering is redundancy produced by gene duplicates [3,4]. Indeed, gene duplication is a major factor shaping prokaryotic and eukaryotic genomes [5-7]. Duplicate genes diverge in their sequence and function [7] and may or may not have the ability to buffer for loss of the respective homolog. While processes other than buffering by duplicates play important roles in robustness against gene loss, e.g. use of alternative pathways [8,9], the relationship between essentiality and the existence of gene duplicates has attracted much attention, and previous work revealed an intricate picture.

For example, estimates of the role of duplicates as backups for gene loss vary widely within and across organisms. Most yeast genes are non-essential, i.e. dispensable, in rich medium or under standard laboratory conditions (>80%, ref. [10]). A study by Gu et al. attributes 23–59% of the dispensability (or survival) to buffering by gene duplicates [11], whereas other studies quote a much lower range (15–28%) [8,12-15]. Only 2% of gene pairs with a synthetic sick or lethal (SSL) mutant phenotype in yeast show detectable similarity [16,17], and amongst the ~20% of mouse genes examined to-date no buffering by duplicates has been observed [18,19].

Several molecular causes may underlie buffering by duplicates, and their relative contributions are still debated. For example, buffering duplicates lack functional redundancy that would be expected from their backup role. Buffering duplicates in yeast have only partially overlapping expression [20] and genetic interaction profiles [13], suggesting their functions have diverged. Alternative explanations for the bias against duplicates amongst essential genes have been suggested. For example, it may be disadvantageous for the cell to retain duplicates for genes with severe (lethal) knockout phenotypes because this may disrupt their finely balanced expression dosage [21]. Further, the correlation between gene expression levels and existence of duplicates suggests buffering for gene loss may only be a by-product of processes that retain duplicates for dosage amplification [12,13,22,23].

Despite the availability of several large-scale datasets on single gene knockouts (KO) or knock-downs (KD) as well as double gene-KOs for all of these organisms, previous studies mainly focused on single organisms like yeast [8,11-14], worm [24] and mouse [18,19]. Major hindrances of a cross-organism comparison are differences in experimental approaches and the specific definition of essentiality used. The types and numbers of essential genes per organism are influenced by several factors: the mutational strategy (insertion, knockout (deletion) or knockdown), growth of the organism in clonal or mixed populations, life cycle stage of the organism, and, for multi-cellular organisms, whether the whole organism or simply a cell line was targeted. Selection pressure is more stringent in mixed than in clonal populations, and we expect lower survival rates in the former. For example, a mutant bacterium of decreased fitness may be selected against in a mixed population, but still be able to form an isolated colony. Insertion experiments may result in leaky expression compared to knockout or deletion experiments, and thus identify fewer essential genes. Finally, while RNAi experiments in worm have reasonably low false-positive and false-negative rates [25,26], we would still expect lower degrees of gene essentiality from this knockdown technique than from gene deletions.

To gain further insights into general principles of buffering by gene duplicates, we conducted a comprehensive cross-organism comparison of essentiality and its relationship to gene duplication, analyzing eleven prokaryotic and eukaryotic organisms – *M. genitalium, H. pylori, H. influenzae, M. tuberculosis, P. aeruginosa, B. subtilis, E. coli, S. cerevisiae *(yeast)*, C. elegans *(worm)*, D. melanogaster *(fly), and *M. musculus *(mouse). To do so, we addressed the above-mentioned challenges in several ways. When selecting essentiality datasets, we aimed to minimize variation in experimental approaches, and, whenever possible, sampled several organisms for a specific technique (Table 1). We tested different definitions of gene duplication, measures of expression levels, and (for yeast) robustness of the results against removal of genes of the whole-gene duplication [27,28] and ribosomal genes (Additional file 1). When assessing the contribution of duplicates to survival upon gene-KO/KD, we normalized by the number of essential genes. Differences in technical approaches certainly influence the extent of essentiality detected amongst organisms; however, if duplicates have a buffering role against loss of gene function then this effect should be observable regardless of the exact number of genes identified to be essential.

**Table 1:** Essentiality and gene duplicates in ten bacterial and eukaryotic organisms.

**Organism**	**Essentiality test**	**No. of tested genes**	**No. of essential genes**	**Number of genes with duplicates (*D *≥ 1)**	**Contribution of duplicates to buffering *C***	***R*^2 ^of *P(S) *vs. Effective family size *D***	***R*^2 ^of *P(S) *vs. E-value**
***M. genitalium***	Random insertion (clones)	460	364	89	-0.13	0.35	0.16

***H. pylori***	Random insertion (population)	1,559	329	358	0.13***	0.26	0.14

***H. influenzae***	Random insertion (population)	1,704	631	400	0.01*	0.27	0.20

***M. tuberculosis***	Random insertion (population)	3,920	614	1,683	0.06***	0.63*	0.40

***P. aeruginosa***	Random insertion (clones)	5,566	364	2,689	0.07***	0.64**	0.80**

***B. subtilis***	Targeted insertion (clones)	4,105	191	1,857	0.0045*	0.37	0.01

***E. coli***	Targeted knockout (clones)	3,221	291	1,940	0.06***	0.64**	0.82**

***S. cerevisiae***	Targeted knockout (clones)	5,318	952	2,531	0.12***	0.00	0.72*

***C. elegans***	Targeted knockdown (clones)	13,915	1,345	9,203	0.09***	0.74**	0.92***

***D. melanogaster***	Targeted knockdown in cell line (clones)	12,145	318	7,004	0.01***	0.00	0.60*

***M. musculus***	Collection of individual experiments	4,267	1,438	3,664	-0.07**	0.03	0.00

The table summarizes properties of the eleven organisms in our analysis, such as (from left to right) the names of the organims; the type of KO/KD experiment; the number of genes *tested *for their essentiality in gene-KO or KD experiments; the number of genes resulting in lethal phenotypes (essential genes); the number of genes with one or more duplicates (*D *≥ 1) amongst the tested genes; the contribution of duplicates to buffering *C = P(S|D ≥ 1)/P(S|D = 0) - 1*; the correlation between *P(S) *and effective family size of the genes *D *(*D *ranges from 0 to 8+, see text); and the correlation between *P(S) *and distance of a gene to its nearest neighbor (measured in -log(E-value), bin size 5). In the experimental descriptions, 'clones' refers to clonal outgrowth on plates or in cultures; 'population' refers to (mixed) population outgrowth in liquid culture. P-value thresholds of 0.05, 0.01, and 0.001 are marked with *, **, and ***, respectively.

KD – knockdown; KO – knockout; *P(S) *– probability of survival; *D *– effective gene family size (number of additional gene duplicates)

Our study reveals heterogeneity of essentiality and the contribution of duplicates to survival that goes beyond what is accountable for by technical differences. We show that organismal complexity and lifestyle, gene function, function similarity, sequence similarity or the number of duplicates per gene are only weak predictors of the buffering capacity – gene expression levels and related measures are the strongest correlates. Simple relationships with respect to essentiality and gene duplication hold true for some organisms, but not for others. Buffering by duplicates plays a significant but small and heterogeneous role.

## Results and discussion

### The extent of essentiality varies widely amongst organisms

If duplicate genes play a significant role in buffering against mutations, then genes with one or more paralogs should have higher chances of survival upon deletion than singletons. This simple relationship has been demonstrated for yeast [11] and *C. elegans *[24], but not yet for other organisms. To test the generality of this prediction, we estimated families of homologous genes for eleven bacterial and eukaryotic organisms based on a BLAST [29] sequence similarity search (E-value < 1.0e-10), and compared survival upon knockout (KO) or knockdown (KD) of genes from these gene families to survival upon KO/KD of singletons (Table 1). We estimate gene expression levels by use of the Codon Bias Index (Methods).

We define the effective family size *D *of a target gene as the number of duplicates remaining after KO or KD. *D = 0 *denotes singletons genes; *D *≥ 1 denotes genes with paralogs. The probability *P(D ≥ 1) *is derived from the fraction of genes in a genome which do have one or more duplicates (paralogs). We also use the probability *P(S) *which describes for an organism chances of survival upon gene-deletion; *P(S) *is derived from the fraction of genes identified as dispensable (non-essential) in large-scale screens. When discussing 'buffering by duplicates' we mean the enrichment of duplicates amongst non-essential genes as inferred from statistical analysis. 'Essentiality/non-essentiality (survival)' is purely based on outcomes of experiments.

**Figure 1:**
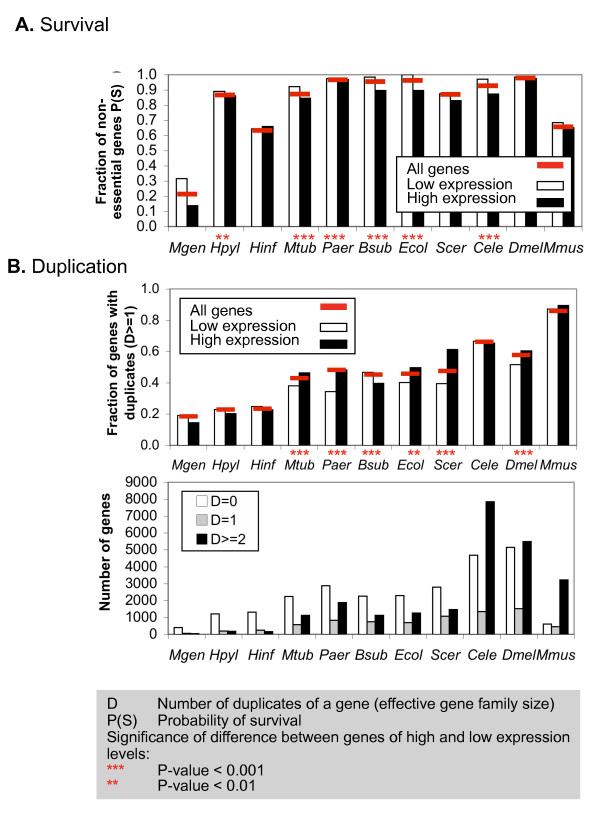
**Chances of survival upon gene-KO/KD vary between organisms**. While the number and fraction of duplicate genes increases from prokaryotes to single- and multi-cellular eukaryotes, the fraction of essential genes (and hence chances of survival upon gene-KO/KD) vary widely. The three panels show the probability of survival *P(S)*(A), the gene family distribution and number of genes with duplicates (*D *≥ 1)(B). Singleton genes are labeled *D = 0*, members of two-gene families are labeled *D = 1*, members of larger gene families are labeled *D *≥ 2. Red bars indicate values for all genes, as also listed in Table 1. High (black) and low (white) gene expression levels are estimated by codon bias indices (see methods). Significant differences between genes of high and low expression (χ^2 ^test) are marked with ** (P-value ≤ 0.01) and *** (P-value ≤ 0.001). *D *– effective gene family size (number of additional duplicates of a gene); *S *– survival upon gene deletion (1-essentiality). *Mgen *– *Mycoplasma genitalium*; *Hpyl *– *Helicobacter pylori*; *Hinf *– *Haemophilus influenzae*; *Mtub *– *Mycobacterium tuberculosis*; *Paer *– *Pseudomonas aeruginosa*; *Bsub *– *Bacillus subtilis*; *Ecol *– *Escherichia coli*; *Scer *– *Saccharomyces cerevisiae *(yeast); *Cele *– *Caenorhabditis elegans *(worm); *Dmel *– *Drosophila melanogaster *(fly); *Mmus *– *Mus musculus *(mouse).

**Figure 2:**
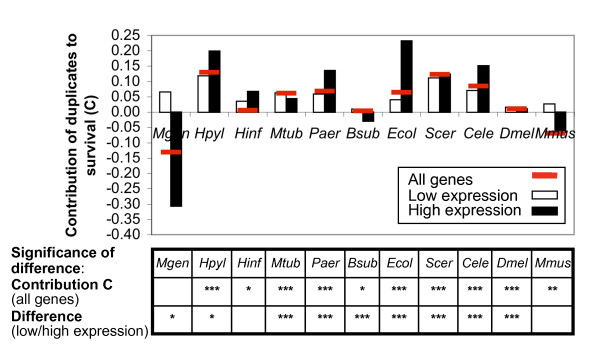
**Small but significant buffering of duplicate genes against gene-KO/KD**. In most organisms of our analysis, duplicates contribute significantly to survival against gene-KO/KD (P-value ≤ 0.05), although to only a small extent. Buffering is increased amongst genes of high expression levels (high CBI, black bars) compared to genes of lower expression levels (white bars). In highly expressed genes, duplicates contribute to survival by up to 23% (*E. coli*). Significant enrichment of duplicates amongst non-essential genes (hypergeometric distribution) and significant differences between genes of high and low expression (χ^2 ^test) are marked with *, **, and *** for P-value thresholds of 0.05, 0.01, and 0.001, respectively. *For abbreviations see Figure 1*.

Table 1, Figure 1 and 2 summarize our results with respect to survival and gene duplication across whole genomes. Most genomes in our dataset have relatively few essential genes; chances for survival upon loss of a single gene are high in both prokaryotes and eukaryotes (*P(S) *> 0.80), except for *M. genitalium, H. influenzae *and mouse (Figure 1A). Genes of high expression levels are more likely to be essential than genes of low expression levels (smaller *P(S)*); in half (six) of the organisms the difference is significant (P-value ≤ 0.01).

In accordance with the expectation that more complex organisms tend to have more duplicate genes, the fraction of genes with duplicates (*D *≥ 1) increases from *M. genitalium *and the other bacteria, to yeast and the three animals (Figure 1B). Compared to other organisms, mouse has a noticeable depletion of singleton genes (*D = 0*) relative to genes with duplicates. In five organisms, there is a significant increase in the fraction of duplicates (*D *≥ 1) amongst highly expressed genes compared to other genes (P-value ≤ 0.01); an exception is *B. subtilis *in which the trend is inverted. When using Codon Adaptation Index or experimental expression data we obtain similar results (Additional file 1).

### Duplicates increase chances of survival – in some organisms more than in others

To assess the contribution of duplicates to survival following gene-KO/KD we define the buffering capacity *C *as *C = P(S|D ≥ 1)/P(S|D = 0) – 1*, where *P(S|D = 0) *is the probability of survival given the gene does not have additional duplicates, i.e. is a singleton. *P(S|D ≥ 1) *is the probability of survival given the gene has one or more additional duplicates. *C *is calculated for each organism and quantifies the increase in probability of survival upon gene-KO/KD for genes which have a duplicate in the genome.

In nine of the eleven organisms, duplicates contribute significantly and positively to survival (P-value ≤ 0.05); with contributions ranging from 1 to 13% (Table 1, Figure 2). The exceptions are *M. genitalium *and mouse in which duplicates appear to decrease chances of KO survival. The extent of buffering by duplicates, i.e. the value of *C*, does not correlate with the organisms' complexity or genome size. Total *C *is largest in yeast, worm and *H. pylori *and smallest in *H. influenzae, B. subtilis *and fly. While the total number and fraction of genes with duplicates increases from simpler to more complex organisms (Figure 1B), the propensity of duplicates to buffer against gene loss varies independently.

Next we ask whether amongst genes with duplicates chances for buffering upon gene loss increase with high expression levels compared to low expression levels. In most of the organisms, there are significant differences in buffering capacity *C *amongst genes of low and high expression levels (P-value ≤ 0.05). However, only in five organisms (*H. pylori*, *P. aeruginosa*, *E. coli*, yeast, and worm), genes of high expression levels and with duplicates have significantly improved chances of survival; with *C *reaching 23% in *E. coli*. In *M. genitalium *and *M. tuberculosis, C *is positive amongst highly expressed genes when examining experimental expression data (Additional file 1); in *B. subtilis *and fly survival is generally very high and a distinction between genes of high or low expression does not have any effect.

These results are robust to various methods of paralog estimation, although exact numbers change depending on parameter settings. We tested, for example, different E-value cutoffs, different length requirements on the match region or when using methods of homology estimation that are completely independent of particular E-value thresholds (Additional file 1).

### Further correlates of buffering capacity

Assuming that paralogs can take over the function of a deleted gene, one may hypothesize that chances of doing so increase i) with the number of paralogs present, and ii) their similarity to the mutant protein. We tested these predictions in the eleven organisms.

**Figure 3:**
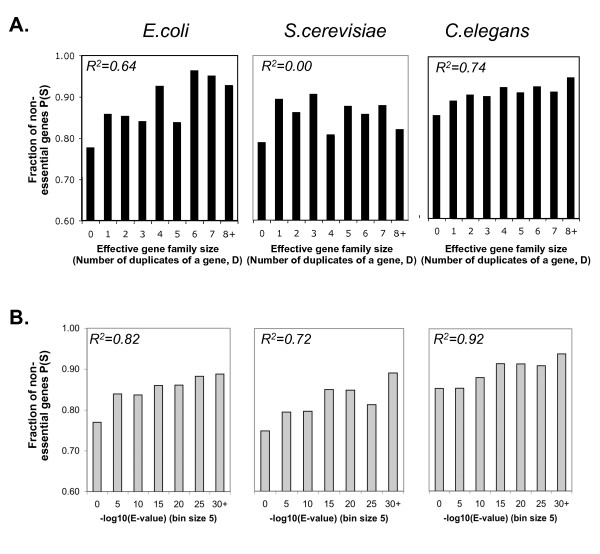
**Survival upon single gene-KO/KD is correlated with the number of duplicates present and their distance to the gene only in some organisms**. For *E. coli*, yeast and worm, we deconvolute the set of duplicates into different effective family sizes (A), or according to the distance with respect to sequence between the deleted gene and its nearest homolog (B). In *E. coli *and worm, chances of survival increase slightly with an increasing number of duplicates present per gene (*D*) or increasing sequence similarity (as measured by the *E-value*). Yeast has no correlation between the effective family size and survival (A), but chances for survival are higher in two-gene families (*D = 1*) than in larger families (*D *≥ 2). *For abbreviations see Figure 1*.

Only in three organisms, *P. aeruginosa, E. coli*, and worm, chances of survival correlate significantly (P-value ≤ 0.05) with both the number of duplicates available per gene and with the distance of the gene to the nearest homolog (*R*^2 ^≥ *0.64 *and *R*^2 ^≥ *0.80*, respectively; Table 1). These correlations have been observed previously in worm [24], but are not common amongst the organisms of our study. Yeast has a decent correlation with distance to the nearest homology (*R*^2^* = 0.72*), but not with the number of duplicates per gene. These results do not change even when removing ribosomal genes or gene pairs originating from the whole-genome duplication [28], or when focusing on highly expressed genes (Additional file 1). Yeast is particularly enriched in two-gene families (*D = 1*) which buffer for each other (Additional file 1). Figure 3A shows these distributions for *E. coli*, yeast and worm.

We further tested *C *for genes in different groups of gene function, without finding strong biases (Additional file 1).

### Two-gene families as model for buffering by duplicates

To better understand buffering by duplicates, we compared the properties of a subset of duplicates which are likely to buffer for each other's function to those which do not buffer for each other. In particular, we analyzed two-gene families which had been tested for both single- and double gene-KOs. Of course, members of larger gene families can also buffer for each other – however, it is more difficult to distinguish buffering genes from those with other functions. For two-gene families, if the double-KO of two non-essential genes is lethal, the two genes are likely to buffer for each other's function in single-KOs, i.e. we call them *buffering duplicates*. Despite the generally low contribution of duplicates to survival upon gene knockout, these two-gene families are paramount candidates for buffering. If a double-KO is viable, reasons other than the presence of a duplicate should explain their viable single-KO phenotype. We call these pairs *non-buffering duplicates*.

Amongst the ~300,000 yeast gene pairs tested for double-KO phenotypes tested in large- and small-scale screens [30], we identified 50 two-gene families with genetic interactions (buffering) and eight two-gene families with a viable double-KO phenotype (non-buffering). These two-gene families represent prime candidates for comparing characteristics of buffering and non-buffering duplicates, respectively. Table 2 and Additional file 1 describe their properties tested across and between the genes. There are also another 551 two-gene families in yeast which have not been tested in double-KO experiments; Additional file 1 describes their characteristics.

**Table 2:** Characteristics of buffering and non-buffering yeast two-gene families

**Feature**	**Source**	**Buffering gene pair – average**	**Buffering gene pair – count**	**Non-buffering gene pair – average**	**Non-buffering gene pair – count**	**t-score**
**Across genes**						

**mRNA abundance (molecules/cell)**	[67]	4.948	91	0.906	14	4.04*

**Protein abundance (molecules/cell)**	[67]	35040	29	2116	4	2.84

**Molecular weight (Da)**	[66]	66299.9	99	91885.0	16	-2.33

**Codon Adaptation Index**	[66]	0.232	99	0.134	16	4.97*

**Codon Bias Index**	[66]	0.187	99	0.051	16	5.18*

**Protein production rate (s^-1^)**	[68]	0.632	90	0.056	12	3.45*

**Proteins produced per mRNA**	[68]	5.733	85	1.388	11	4.07*

**Transcription rate (s^-1^)**	[68]	0.109	85	0.040	11	2.87

**Protein half-life (min)**	[69]	108.5	74	177.1	13	-0.50

**dN/dS**	[70]	0.056	56	0.113	8	-1.95

**No. orthologs in 14 organisms**	[32]	8.1	94	5.8	15	1.52

**No. protein-protein interactions**	[71]	15.2	84	4.3	14	4.50*

**Between genes**						

**Sequence similarity (%)**	BLAST output	54.3	50	32.5	8	4.91*

**Shortest path – Functional network**	[31]	1.27	48	1.63	8	-1.26

**Vector similarity – Functional interactions**	[31]	0.15	23	0.04	7	2.04

**Vector similarity – Physical interactions**	[30]	0.13	25	0.03	8	2.01

**Vector similarity – Genetic interactions**	See methods	0.01	26	0.07	7	-1.49

**Vector similarity – KO phenotypes**	[63]	0.17	10	0.11	2	0.27

**Worm two-gene families (subset)**						

**Length (nt)**	[66]	1556	254	1359	32	1.10

**Codon Adaptation Index**	[64]	0.396	254	0.326	32	2.46

**dN (Ka)**	Analysis by [33]	0.34		0.50		

The table lists a selection of characteristics tested for the two sets of buffering and non-buffering yeast two-gene families, respectively. Also see **Table 3 **for description of the data. A small number of characteristics could also be tested for worm two-gene families, identified in published work [33]. Due to multiple hypothesis testing, a t-score > 3.26 should be considered significant at an adjusted P-value of 0.05 (Bonferroni); significant scores are marked with *. An E-value of '0' signifies an E-value that is smaller than 10^-360^.

Both buffering and non-buffering two-gene families are defined by the same E-value threshold (10^-10^, Methods); however, buffering genes have significantly higher sequence identity between the members (P-value < 0.05; Table 2). Buffering genes are also more conserved than non-buffering genes, i.e. have slower rates of evolution and more orthologs across organisms.

We examined the functional similarity between genes in the sets of pairs, testing whether buffering duplicates are more similar in their function than non-buffering duplicates. We find that genes buffering two-gene families have mostly identical function descriptions, and descriptions for non-buffering genes are similar but not identical (Table 3, 4) – however, this finding is only qualitative. To quantify functional distance, we measured the average shortest path between the genes in a network of functional relationships [31]: buffering genes had slightly shorter paths between each other than non-buffering genes (not significant, Table 2), i.e. their functions are closer to each other. Other quantitative measures of gene function can be derived from the number and types of physical protein-protein interactions, functional interactions [31], genetic interactions or gene-KO phenotypes under various conditions. Buffering genes are more similar to each other than non-buffering genes in all these measures except for genetic interactions, although the trends are not significant (Table 2). The lack of similarity of genetic interaction profiles between buffering genes is consistent with recent findings by Ihmels et al. [13] although these authors included epistatic interactions other than lethal double-KO phenotypes in their analysis.

**Table 3:** Examples of yeast buffering two-gene families (SSL double-KO phenotype)

**Name**	**Function**	**Name**	**Function**	**E-value**	**Sequence identity (%)**
**YIL159W BNR1**	Formin, nucleates the formation of linear actin filaments, involved in cell processes such as budding and mitotic spindle orientation which require the formation of polarized actin cables, functionally redundant with BNI1	**YNL271C BNI1**	Formin, nucleates the formation of linear actin filaments, involved in cell processes such as budding and mitotic spindle orientation which require the formation of polarized actin cables, functionally redundant with BNR1	1E-82	32

**YML075C HMG1**	One of two isozymes of HMG-CoA reductase that catalyzes the conversion of HMG-CoA to mevalonate, which is a rate-limiting step in sterol biosynthesis; localizes to the nuclear envelope; overproduction induces the formation of karmellae	**YLR450W HMG2**	One of two isozymes of HMG-CoA reductase that convert HMG-CoA to mevalonate, a rate-limiting step in sterol biosynthesis; overproduction induces assembly of peripheral ER membrane arrays and short nuclear-associated membrane stacks	0	62

**YKR067W GPT2**	Glycerol-3-phosphate acyltransferase located in both lipid particles and the ER; involved in the stepwise acylation of glycerol-3-phosphate and dihydroxyacetone, which are intermediate steps in lipid biosynthesis	**YBL011W SCT1**	Glycerol 3-phosphate/dihydroxyacetone phosphate dual substrate-specific sn-1 acyltransferase of the glycerolipid biosynthesis pathway, prefers 16-carbon fatty acids, similar to Gpt2p, gene is constitutively transcribed	2E-118	36

**YEL042W GDA1**	Guanosine diphosphatase located in the Golgi, involved in the transport of GDP-mannose into the Golgi lumen by converting GDP to GMP after mannose is transferred its substrate	**YER005W YND1**	Apyrase with wide substrate specificity, involved in preventing the inhibition of glycosylation by hydrolyzing nucleoside tri- and diphosphates which are inhibitors of glycotransferases; partially redundant with Gda1p	5E-28	27

**YKL020C SPT23**	ER membrane protein involved in regulation of OLE1 transcription, acts with homolog Mga2p; inactive ER form dimerizes and one subunit is then activated by ubiquitin/proteasome-dependent processing followed by nuclear targeting	**YIR033W MGA2**	ER membrane protein involved in regulation of OLE1 transcription, acts with homolog Spt23p; inactive ER form dimerizes and one subunit is then activated by ubiquitin/proteasome-dependent processing followed by nuclear targeting	1E-163	37

**YGR038W ORM1**	Evolutionarily conserved protein with similarity to Orm2p, required for resistance to agents that induce the unfolded protein response; human ortholog is located in the endoplasmic reticulum	**YLR350W ORM2**	Evolutionarily conserved protein with similarity to Orm1p, required for resistance to agents that induce the unfolded protein response; human ortholog is located in the endoplasmic reticulum	3E-68	72

**YER087C-B SBH1**	Beta subunit of the Sec61p ER translocation complex (Sec61p-Sss1p-Sbh1p); involved in protein translocation into the endoplasmic reticulum; interacts with the exocyst complex	**YER019C-A SBH2**	Ssh1p-Sss1p-Sbh2p complex component, involved in protein translocation into the endoplasmic reticulum	8E-19	55

**YHL003C LAG1**	Ceramide synthase component, involved in synthesis of ceramide from C26(acyl)-coenzyme A and dihydrosphingosine or phytosphingosine, functionally equivalent to Lac1p	**YKL008C LAC1**	Ceramide synthase component, involved in synthesis of ceramide from C26(acyl)-coenzyme A and dihydrosphingosine or phytosphingosine, functionally equivalent to Lag1p	6E-169	73

**YHR066W SSF1**	Constituent of 66S pre-ribosomal particles, required for ribosomal large subunit maturation; functionally redundant with Ssf2p	**YDR312W SSF2**	Protein required for ribosomal large subunit maturation, functionally redundant with Ssf1p	0	94

**YPR159W KRE6**	Protein required for beta-1,6 glucan biosynthesis; putative beta-glucan synthase; appears functionally redundant with Skn1p	**YGR143W SKN1**	Protein involved in sphingolipid biosynthesis; type II membrane protein with similarity to Kre6p	0	68

Two-gene families and their phenotypes in double-KOs are a good model for buffering by gene duplicates. We distinguish between 'buffering genes' (50), i. e. gene pairs resulting in a synthetic sick or lethal (SSL) phenotype upon double-KO; and 'non-buffering genes' (eight), i. e. gene pairs that result in a viable phenotype upon double gene-KO, and which are thus unlikely to buffer for each other in single gene-KO.

Tables **3 **and **4 **list the functions of a subset of buffering and all eight non-buffering gene pairs, respectively, with one pair per row. The ten buffering gene pairs in this table originate from the same large-scale screens as the eight non-buffering pairs in table **4**. The remaining 40 buffering gene pairs originate from small-scale screens, and are listed in the Additional file 2. The descriptions of functions are taken from SGD [66]. Buffering genes (this table) are more often described as having identical functions than non-buffering genes (**Table 4**).

**Table 4:** Examples of yeast non-buffering two-gene families (viable phenotype in double-KO)

**Name**	**Function**	**Name**	**Function**	**E-value**	**Sequence identity (%)**
**YJR075W HOC1**	Alpha-1,6-mannosyltransferase involved in cell wall mannan biosynthesis; subunit of a Golgi-localized complex that also contains Anp1p, Mnn9p, Mnn11p, and Mnn10p; identified as a suppressor of a cell lysis sensitive pkc1-371 allele	**YGL038C OCH1**	Mannosyltransferase of the cis-Golgi apparatus, initiates the polymannose outer chain elongation of N-linked oligosaccharides of glycoproteins	2E-40	27

**YGR188C BUB1**	Protein kinase that forms a complex with Mad1p and Bub3p that is crucial in the checkpoint mechanism required to prevent cell cycle progression into anaphase in the presence of spindle damage, associates with centromere DNA via Skp1p	**YJL013C MAD3**	Component of the spindle-assembly checkpoint complex, which delays the onset of anaphase in cells with defects in mitotic spindle assembly; interacts physically with the spindle checkpoint proteins Bub3p and Mad2p	2E-50	35

**YHR119W SET1**	Histone methyltransferase, subunit of the COMPASS (Set1C) complex which methylates histone H3 on lysine 4; required in transcriptional silencing near telomeres and at the silent mating type loci; contains a SET domain	**YJL168C SET2**	Histone methyltransferase with a role in transcriptional elongation, methylates a lysine residue of histone H3; associates with the C-terminal domain of Rpo21p; histone methylation activity is regulated by phosphorylation status of Rpo21p	2E-16	30

**YDR528W HLR1**	Protein involved in regulation of cell wall composition and integrity and response to osmotic stress; overproduction suppresses a lysis sensitive PKC mutation; similar to Lre1p, which functions antagonistically to protein kinase A	**YCL051W LRE1**	Protein involved in control of cell wall structure and stress response; inhibits Cbk1p protein kinase activity; overproduction confers resistance to cell-wall degrading enzymes	5E-34	34

**YJR131W MNS1**	Alpha-1,2-mannosidase involved in ER quality control; catalyzes the removal of one mannose residue from Man9GlcNAc to produce a single isomer of Man8GlcNAc in N-linked oligosaccharide biosynthesis; integral to ER membrane	**YHR204W MNL1**	Alpha mannosidase-like protein of the endoplasmic reticulum required for degradation of glycoproteins but not for processing of N-linked oligosaccharides	9E-25	25

**YDR420W HKR1**	Serine/threonine rich cell surface protein that contains an EF hand motif; involved in the regulation of cell wall beta-1,3 glucan synthesis and bud site selection; overexpression confers resistance to Hansenula mrakii killer toxin, HM-1	**YGR014W MSB2**	Mucin family member at the head of the Cdc42p- and MAP kinase-dependent filamentous growth signaling pathway; also functions as an osmosensor in parallel to the Sho1p-mediated pathway; potential Cdc28p substrate	6E-12	29

**YML061C PIF1**	DNA helicase involved in telomere formation and elongation; acts as a catalytic inhibitor of telomerase; also plays a role in repair and recombination of mitochondrial DNA	**YHR031C RRM3**	DNA helicase involved in rDNA replication and Ty1 transposition; relieves replication fork pauses at telomeric regions; structurally and functionally related to Pif1p	5E-102	40

**YJL092W HPR5**	DNA helicase and DNA-dependent ATPase involved in DNA repair, required for proper timing of commitment to meiotic recombination and the transition from Meiosis I to Meiosis II; potential Cdc28p substrate	**YOL095C HMI1**	Mitochondrial inner membrane localized ATP-dependent DNA helicase, required for the maintenance of the mitochondrial genome; not required for mitochondrial transcription; has homology to E. coli helicase uvrD	2E-18	21

See **Table 3 **for description. Tables **3 **and **4 **list the functions of a subset of buffering and all eight non-buffering gene pairs, respectively, with one pair per row. The descriptions of functions are taken from SGD [66]. Buffering genes (**Table 3**) are more often described as having identical functions than non-buffering genes (this table).

Buffering and non-buffering genes show clear differences in terms of transcriptional and translational regulation (Table 2). Buffering genes have higher mRNA and protein expression levels. Measures of translation efficiency, e.g. protein length, molecular weight, Codon Adaptation Index (CAI), or protein production rate, are significantly elevated in buffering genes compared to non-buffering ones (P-value ≤ 0.05); protein degradation is slightly decreased. Interestingly, some of these measures (e. g. length, CAI) are significantly more different between members of a buffering gene pair than between members of a non-buffering gene pair (Additional file 1).

We also extracted orthologs of the buffering and non-buffering yeast two-gene families in fly, worm and mouse using InParanoid [32]. (None of the yeast genes had orthologs in *E. coli*). If a buffering gene pair in yeast has a single-gene ortholog in another organism (without additional duplicates), we expect this ortholog to be essential – more often than single-gene orthologs of non-buffering gene pairs. If an ortholog of a buffering two-gene family has paralogs, we do not expect it to be essential. Indeed, buffering gene pairs are enriched for essential single orthologs compared to non-buffering gene pairs, although the trend is very weak and not significant due to small numbers in the dataset (Table 5, P-value = 0.19; Additional file 1, P-value = 0.07). There are several examples of essential single orthologs of buffering gene pairs: HMG1 and HMG2 are isozymes of HMG-CoA reductase in yeast (Table 3) and their double KO phenotype is lethal. The genes have one ortholog in worm (F08F8. 2) and one in mouse (HMG-CoAR, MGI96159) which both have embryonic lethal KO/KD phenotypes. SSF1 and SSF2 are yeast proteins required for ribosomal large subunit maturation (Table 3), and they have single essential orthologs in worm (K09H9. 6, lpd-6) and fly (CG5786, Peter Pan).

**Table 5:** Orthologs of yeast buffering and non-buffering two-gene families

	**Buffering pairs**	**Non-buffering pairs**
**Single-gene ortholog in fly, worm or mouse (no duplicate)**		

- essential	11	0

- non-essential	13	3

**Multi-gene orthologs in fly, worm or mouse (with duplicates)**		

- all duplicates essential	1	0

- all duplicates non-essential	6	0

**Other (mix of the above or no information)**		

	24	6

This table lists the number of instances in which for the buffering and non-buffering yeast two-gene families, respectively, single or multiple orthologs were found in fly, worm or mouse and their KO-phenotype if known. Also see **Table 3 **for description of the data. Orthologs are divided into single-gene orthologs (no additional homologs in the organism) and multi-gene orthologs (additional paralogs). Single- or multi-gene orthologs can be essential or non-essential in the other organism.

For further validation, we extracted the 143 worm two-gene families tested in double-RNAi knockdowns [33] which consist of 16 pairs of synthetic sick or lethal (SSL) phenotypes, i.e. buffering duplicates, and 127 non-buffering duplicate gene pairs. Unfortunately, there are no experimental data available for worm genes to test for measures of transcriptional and translational efficiency. When calculating CAI for the worm sequences, we found a significant bias confirming the trend in yeast (Table 2). Buffering genes are more efficiently translated than non-buffering genes.

Noticeably, yeast is enriched for buffering gene pairs (50) vs. non-buffering gene pairs (eight) compared to worm (16 and 143-16 = 127, respectively). This bias holds true even if only regarding the yeast gene pairs identified in large-scale screens: ten buffering and eight non-buffering pairs. Previous work has shown that yeast is enriched for buffering gene pairs which originate from the whole genome duplication [34]. In addition, RNAi-based screens in worms may miss synthetically lethal interactions and thus have a high false-negative rate amongst gene pairs found to be non-buffering.

## Conclusion

Our study provides a systematic and semi-quantitative assessment of essentiality and gene duplication across eleven prokaryotic and eukaryotic organisms revealing a heterogeneous picture. To the best of our knowledge, this is the first such organism-wide comparison.

Chances of survival upon gene deletion are very high in most organisms (>80%), i.e. there are only few essential genes (Figure 1A). We observe some variation in survival that cannot be explained by experimental differences alone. The bacteria in our dataset have been analyzed come from different experimental backgrounds (i.e. insertion vs. deletion, population vs. clonal study, Table 1). For example, screens of mixed populations with random gene insertions identify more essential genes than clonal studies, e.g. *H. pylori*, *H. influenzae*, and *M. tuberculosis *vs. *P. aeruginosa, B. subtilis *and *E. coli *(Table 1); however, there is no general trend.

The extremely high chances of survival in fly (Figure 1A) can be (in part) attributed to the use of a cell line rather than the whole organism and of RNAi knockdowns instead of full gene deletion [35], and may be an underestimate due to current technical limitations. However, in worm, the same technique, RNAi-KDs, on the whole organism also produced high survival rates, but a much higher contribution of duplicates to survival (see below).

The low chances of survival in mouse are likely due to the mouse dataset not originating from a large-scale screen, but from individual experiments that may have preferentially targeted and reported essential genes. For example, the gene targets in the mouse dataset are strongly enriched for orthologs of human disease genes (OMIM data, *not shown*); thus the dataset is biased. The lack of buffering by duplicate genes in mouse has been demonstrated recently [18,19]; however, with the availability of an unbiased large-scale essentiality screen in mouse these results may be refined.

The degree of gene essentiality (or degree of survival) can be influenced by the experimental technique and the definition of essentiality that is used. In contrast, if duplicates contribute to survival upon gene loss, then this effect should be detectable irrespective of the number of essential and non-essential genes identified (provided that the selection is unbiased). In other words, we expect buffering by duplicates to be less dependent on technical differences than essentiality alone. We introduced statistical tests to assess the significance of buffering by duplicates (Figure 2). A small P-value implies that duplicates are significantly enriched amongst non-essential genes compared to random and *vice versa*. Thus, for example, *H. pylori *has only few genes with duplicates (Figure 1B), but these duplicates exhibit a significant contribution to survival upon gene knockout (Figure 2). Likewise, *B. subtilis *and *E. coli *have similar degrees of gene essentiality (one examined by insertion, the other by knockout experiments), and similar fractions of duplicate genes, but very different contributions of these duplicates to survival.

Duplicates significantly and positively contribute to survival in nine of the eleven organisms, but have noticeable effects only in six (>5%; *H. pylori*, *M. tuberculosis*, *P. aeruginosa*, *E. coli*, yeast, worm; Figure 2). Given that duplicates make up to 80% of eukaryotic genomes (Figure 1B), the small contribution is surprising and points to dominant roles of other buffering processes, such as rerouting metabolic flux (see ref. [9] for an example).

Buffering by duplicates is uncorrelated with organismal complexity. Buffering capacity varies widely amongst bacteria and eukaryotes, even when accounting for differences in experimental approaches (Table 1). *M. genitalium, H. influenzae, B. subtilis*, fly and mouse show low or even negative contributions of duplicates to buffering; *H. pylori*, yeast and worm show the highest. *M. genitalium *is a parasite with a small range of host- or tissue-specific living conditions [36] and a very small genome [37](Figure 1). Its low rate of survival upon gene-KO could be explained by the low number of duplicate genes and the lack of condition-specific dispensability of genes which boost survival rates under normal conditions [12]. However, the same reasoning could apply to *H. pylori *and *H. influenzae *which have genome sizes similar to *M. genitalium *and restricted living conditions, but have much higher survival rates and different buffering capacities of duplicates. Mouse represents an exception in the analysis by having relatively low survival rates (Figure 1A), a higher ratio of duplicates vs. singletons than other organisms (Figure 1B), but a negative contribution of duplicates to survival (Figure 2). As explained above, conclusions in mouse may be refined later.

Next we examined gene characteristics which have been suggested to influence buffering capacity. For example, we would expect duplicates of high sequence proximity (measured by E-value) to be more likely to buffer for loss of function than duplicates that diverged in their sequence. Similarly, we would expect genes with many duplicates (large gene families) to be more likely to be buffered for loss of function than genes of small families. Both expectations are fulfilled in only some of the organisms (Table 1), e.g. in the two most thoroughly studied organisms yeast and worm, but not in others.

Related to sequence similarity is function, which is more dissimilar amongst buffering duplicates than naively expected, when measured in terms of expression regulation [20] and genetic interactions [13]. When evaluating function similarity in terms of verbal descriptions, shortest path length in a network of functional relationships, and in terms of similarity of their KO-phenotype and physical interaction vectors, buffering genes were slightly (but not significantly) more similar to each other in function than non-buffering genes (Table 2). Thus, function similarity is also only a weak indicator of buffering capacity of duplicates.

The single best correlate of buffering capacity by gene duplicates (identified in our study) is expression level. Genes of high expression levels tend to have more duplicates, but these duplicates are also more likely to buffer for loss of the gene's function. (Note the subtle difference between the two observations.) The trend holds true for all organisms with positive buffering capacity (except for *M. tuberculosis*) and for different measures of expression levels (Additional file 1). For example, in highly expressed genes in *E. coli, C *increases to 23%. Likewise, buffering two-gene families in yeast have higher mRNA and protein abundance than non-buffering two-gene families, higher transcription and translation rates and smaller protein degradation rates (Table 2).

In sum, buffering by gene duplicates only plays a significant and visible role in robustness against gene loss in some organisms but not in others. Factors influencing such buffering are, in decreasing order of approximate importance, gene expression levels, sequence distance between duplicates, the number of duplicates available per gene, the gene's function and the type of organism and its lifestyle. Such ranking holds true despite differences in experimental approaches. The lack of consistency across organisms, lack of strong correlates and low extent of buffering by duplicates suggests that buffering by duplicates is indeed merely a by-product of other processes. Genes with high expression levels are more likely to be essential [38] and have increased duplicate retention rates [12,23]. These duplicates thus likely function to amplify gene dosage [22], which is supported by their tendency to be co-expressed [13]. Our analysis shows that only in relatively few cases these duplicates serve as backup for the loss of gene function.

## Methods

### Data sets

We obtained the amino acid sequences for ten genomes (*Mycoplasma genitalium; Bacillus subtilis; Helicobacter pylori; Haemophilus influenzae; Mycobacterium tuberculosis; Pseudomonas aeruginosa; Escherichia coli; Saccharomyces cerevisiae *(yeast)*; Caenorhabditis elegans *(worm)*; Drosophila melanogaster *(fly); *Mus musculus *(mouse)) from a collection in the SUPERFAMILY database [39]. Information on gene essentiality (lethal phenotypes upon single gene-KO or KD) was taken from publications [25,35,36,40-46]. Table 1 provides an overview of the number of genes in tested each organism (background set) and the number of genes identified to be essential. The table describes briefly the experimental strategy, as described in the publications and in the SEED database . All screens were conducted in rich medium and on whole organisms except for fly (cell line). For mouse, data of ~4,000 individual knockout experiments were obtained from the Mouse Genome Database [47].

To-date, large-scale double-KO/KD data is only available for yeast and worm. For yeast we compiled in addition to the original data published by Tong et al. [16,48] 13 datasets identified as 'systematic screens' in the BioGRID database [30,49-60]. In a parsimonious approach, we only included data on lethal phenotypes of double-KOs in our study and no other epistatic interactions. To calculate the background set of *tested *gene pairs, we paired the 204 bait genes identified in the 14 analyses with all non-essential yeast genes [42], resulting in ~300,000 tested pairs.

For worm we extracted data from two large-scale double KD screens [26,61], which comprise 52781 tested gene-pairs and 3927 genetic interactions. Another study in worm specifically targeted two-gene families with a single ortholog in yeast [33], and we used these pairs to investigate properties of two-gene families.

### Homology estimation

We measured similarity between all sequences using a BLAST all-against-all search [29], and required an E-value < 10^-10 ^for two genes to be predicted homologs. This E-value threshold was established in yeast and adjusted accordingly in organisms of very different genome size, e.g. in *M. genitatlium *(10^-9^) and worm (3.0*10^-10^). This threshold identified 609 two-gene families in yeast. We tested several other methods of homology prediction including different E-value thresholds, E-value-independent methods and use of InParanoid [32], all with results qualitatively identical to those discussed here (Additional file 1).

### Estimates of gene expression levels

As a surrogate for gene expression levels, we calculated the Codon Bias Index (CBI) for each gene using the CodonW server [62], with standard settings and parameters for the respective organism. We also calculated the Codon Adaptation Index (CAI). However, since it requires a reference dataset of expressed genes (which was not always available) we consider CAI less appropriate of a measure than CBI. Both measures are expected to work less well in multi-cellular organisms due to tissue-specific expression which may not be captured by these sequence features. For further validation, we extracted from literature experimental expression data for all organisms except *H. pylori*. Results for CAI and experimental expression data are in Additional file 1. For the results in Figure 1 and 2, we rank-ordered the CBI values within each genome and selected subsets of genes with the highest or lowest CBI; the sizes of the subsets varied according to the organism's genome size. See Additional file 1 for details.

### Two-gene families and their characteristics

In yeast, 50 two-gene families were identified as buffering (SSL phenotype) and eight two-gene families as non-buffering (viable phenotype). The buffering pairs consist of nine pairs identified in the 14 large-scale double-KO screens (see above), and 42 additional pairs identified in small-scale experiments and listed in BioGRID [30]). The non-buffering pairs originate from pairs tested in 14 large-scale screens and found to have viable phenotypes. Table 2 describes characteristics *between *the two members of a gene family and characteristics of *individual *genes, averaged across the whole set. For vector comparisons, we constructed binary vectors (1 = observation, 0 = no observation) based on networks of functional interactions [31], genetic interactions (see description of datasets above), physical interactions (extracted from BioGRID [30]), and single gene-KO phenotypes [63]. The similarity between two vectors is measured as the percentage of shared positive interactions (Jaccard Index). More results are in Additional file 1.

As a control for the effects of WGD genes, we also compared some characteristics in all 609 yeast two-gene families split into 108 and 501 two-gene families with and without evidence for their origin in the WGD [28], respectively (Additional file 1). As another control, we extracted the 143 worm two-gene families, which were identified and tested by Tischler et al. [33] and calculated codon adaptation indices [64](Additional file 1). Results from these controls are consistent with those from the yeast analysis.

We used the FunSpec server [65] and SGD [66] for yeast protein function annotation. The SUPERFAMILY database [39] was used for annotation of ribosomal proteins in yeast. Genes originating from the whole-genome duplication were taken directly from the published paper [28]. Characteristics described in Table 2 are obtained from the sources quoted in the table and in Additional file 1. For the ortholog analysis described in Table 5, we extracted information from InParanoid [32], and mapped that against the gene essentiality data described above. Information on yeast two-gene families is presented in Additional file 2.

## Abbreviations

CAI: Codon Adaptation Index; CBI: Codon Bias Index; *D*: effective gene family size (number of additional gene duplicates); E-value: expectation value; KD: knockdown; KO: knockout; MIPS: Munich Information Center for Protein Sequences; *P(S)*: probability of survival upon single- or double gene-KO or KD; *R*^2^: squared Pearson correlation coefficient; SGA: Synthetic Genetic Array; SSL: synthetic sick or lethal (mutant); SGD: *Saccharomyces *Genome Database; WGD: whole-genome duplication.

Organisms: *M. genitalium*: *Mycoplasma genitalium*; *H. pylori*: *Helicobacter pylori*; *H. influenzae*: *Haemophilus influenzae*; *M. tuberculosis*: *Mycobacterium tuberculosis*; *Paer*: *Pseudomonas aeruginosa*; *B. subtilis*: *Bacillus subtilis*; *E. coli*: *Escherichia coli*; *S. cerevisiae*: *Saccharomyces cerevisiae *(yeast); *C. elegans*: *Caenorhabditis elegans *(worm); *D. melanogaster*: *Drosophila melanogaster *(fly); *M. musculus*: *Mus musculus *(mouse).

## Authors' contributions

KH conducted the experiments, analyzed results and wrote the paper. EMM provided valuable input and support at all stages of the project. CV initiated and guided the project, conducted some of the experiments, analyzed results and wrote the paper. All authors read and approved the final manuscript.

## Supplementary Material

Additional file 1 Supplementary Notes. Additional figures and comments on the analyses. Click here for file

Additional file 2 Supplementary Data. Data on yeast gene pairs collected during the analyses. Click here for file
