# Supplementary material for: Buffering by gene duplicates: an analysis of molecular correlates and evolutionary conservation
Source: BMC Genomics. 2008 Dec 16;9:609. doi: 10.1186/1471-2164-9-609 (PMC2627895; doi:10.1186/1471-2164-9-609)
Supplement: Additional file 1 — Supplementary Notes. Additional figures and comments on the analyses. [file 1471-2164-9-609-S1.pdf]

The extent of gene essentiality and buffering by duplicates is not conserved across organisms

Supplementary Material

Kevin Hannay[1], Edward M. Marcotte[1], Christine Vogel[1]
[1] Institute for Cellular and Molecular Biology, University of Texas at Austin, 2500 Speedway, MBB 3.210, Austin, TX 78712
*Corresponding author: cvogel@mail.utexas.edu
phone: +1 512 232 3919 fax: +1 512 471 2149

Abbreviations:

CAI – Codon Adaptation Index; *D* – effective gene family size (number of additional gene duplicates); E-value – expectation value; KD – knockdown; KO – knockout; MIPS **-** Munich Information Center for Protein Sequences; *P(S)* – probability of survival upon single- or double-gene KO or KD; R^2 – squared Pearson correlation coefficient; SGA– Synthetic Genetic Array; SSL – synthetic sick or lethal (mutant); SYD – Stanford Yeast Database; WGD – whole-genome duplication

Organisms: *M. genitalium - Mycoplasma genitalium; H. pylori – Helicobacter pylori; H. influenzae – Haemophilus influenzae; M. tuberculosis - Mycobacterium tuberculosis; Paer - Pseudomonas aeruginosa; B. subtilis – Bacillus subtilis; E. coli – Escherichia coli; S. cerevisiae – Saccharomyces cerevisiae* (yeast)*; C. elegans – Caenorhabditis elegans* (worm)*; D. melanogaster – Drosophila melanogaster* (fly); *M. musculus – Mus musculus* (mouse)

Contents

Paralog estimation based on E-value cutoffs 2
Paralog estimation based methods other than E-value cutoffs 3
Experimental expression data 4
Codon Adaptation Index 5

Supplementary Notes

Parameters and Datasets

Unless specified otherwise, the results presented in the **Supplement** use the same data as described in the main text. In our study, we analyzed three different parameters. 1.) The probability of survival $P(S)$ of a cell or organism upon knockout (KO) or knockdown (KD) of a single gene or of two genes. $P(S)$ represents the dispensability of a gene, it is calculated as *1-(fraction of essential genes)* in a set of genes. 2.) The probability of having a duplicate gene, calculated as the fraction of genes with at least one duplicate ($D \geq 1$) in a genome. 3.) The conditional probability $P(S|D=x)$ of survival given a certain number of paralogs. $P(S|D=0)$ signifies survival chances amongst singletons (in single-gene KO/KDs). $P(S|D \geq 1)$ signifies survival chances of genes with at least one duplicate. 4.) The contribution of duplicates to survival of gene KOs/KDs (buffering capacity) $C = P(S|D \geq 1)/P(S|D=0) - 1$.

We ask whether these parameters change depending on the organism of study, the expression levels of genes, and their functions.

1. Estimation of gene duplicates (paralogs)

To validate our prediction of paralogs (gene duplicates), we examined gene family size distributions of the resulting groups of gene duplicates, and we tested several alternative approaches and compared our results to previous studies by Tong et al. [1, 2] and to the gene families obtained using a single E-value cutoff (10^{-10}).

Figure S1. Gene family size distribution for all genomes in our analysis

Gene families are defined by BLAST E-value<10^{-10} (3×10^{-10} for worm, fly, 1×10^{-9} for *Mycoplasma*). (**A**) absolute numbers; (**B**) fraction of genome. The distributions are as expected: small gene families are more frequent than large families. The fraction of gene families (compared to singletons) increases in eukaryotes compared to prokaryotes and in multi-cellular compared to uni-cellular organisms. There is no obvious enrichment of two-gene families ($D=1$) in yeast. These results confirm the validity of our method of paralog estimation.

Paralog estimation based on E-value cutoffs

First, we tested different E-value thresholds for homology estimation in yeast. For all three different E-values (10^{-10}, 10^{-20}, 10^{-30}), we observe a significant enrichment in homologous genes amongst double knockout mutants with an SSL phenotype. When applying the same E-value cutoff used by Tong et al. [1, 2] of 10^{-8}, we obtained ~2% of homologous genes. This result is identical with what Tong et al. report, confirming our estimate. In the paper, we use a threshold of 10^{-10}, as this proved to be the best compromise between conservative homolog estimation (low false-positive rate) but sufficiently many gene families for statistically meaningful analysis.

In addition to use of an E-value threshold, we tested several other constraints on paralog estimation: a) bidirectionality of the hit; b) length of the match region between two alignments; and c) single-linkage clustering. The results of these tests were examined manually with respect to the gene family size distribution, and sequence alignments of selected gene families.

a) We tested whether gene family sizes would change when requiring both genes (query gene and its match) to have their match's E-values below the threshold, instead of just requiring the query gene to have an E-value<threshold to its match. This method represents a stricter paralog definition, decreasing gene family sizes and increasing the number singletons. As this additional requirement did not introduce any obvious advantage we decided against its use.

b) Sequences can match across their whole length or along only part of their length. This behavior is

expressed by the ‘alignment match length’ which denotes the fraction of the shorter sequence that is aligned to the other sequence. We tested match length requirements of 0.6, 0.7 and 0.8, but did not observe significant changes to the gene family size distributions. The E-value itself is partially a function of match length, thus a relatively stringent cutoff (such as 10^{-10}) indirectly requires substantial match length. For our analysis of buffering capacity of homologous genes we did not require the genes to match over their entire length, as even parts of the gene (protein) could buffer for the function of the other.

c) Gene-families can be reconstructed by grouping genes with common paralogs into one family. As our method includes local matches (matches along only part of the sequence, see b), a single-linkage clustering algorithm bears the danger of combining genes, via common homologs, that have no sequence similarity at all and are not paralogs. In addition, for our analysis we were interested in the number of paralogs per gene (effective gene family size *D*) rather than the actual gene family sizes, thus gene family clustering again did not seem a feasible step to do.

In sum, all three variations did not visibly improve our paralog estimation, and in the spirit of parsimony we decided for simple application of one E-value cutoff. Note that, as described in the paper, this E-value cutoff has been adjusted in genomes much smaller or larger than yeast.

Paralog estimation based methods other than E-value cutoffs

We tested additional methods of paralog estimation independent of the absolute E-value between two sequences.

a) In the ‘drop’ method, we examined for each gene the difference in –log(E-value) to its rank-ordered hits, with the minimal -log[E-value]>3. For each gene, we counted a hit as homolog if the difference between its –log[E-value] and the –log[E-value] of the next better hit was smaller than 2, 3 or 5. In other words, we counted all hits as homologs of a particular gene if their -log[E-value]s were similar to each other and significantly better than the -log[E-value]s of all other hits. We produced gene families from these groups of homologs using single linkage clustering. The ‘drop’ method resulted in a similar gene family size distribution as the E-value ‘cutoff’ method.

b) In the ‘bidirectional best hit’ method, gene pairs qualified as paralogs if they had each other as their best hit, independent of the actual E-value, but requiring a minimum –log[E-value]>5. The disadvantage of this method is that it only produces pairs of paralogs, but not larger gene families.

c) For yeast we also estimated paralogs using the Inparanoid database [3]. In this approach, each yeast gene was defined as a paralog of a query gene if its BLAST score is better than the BLAST score to an ortholog of the query gene. Estimating yeast paralogs with *Arabidopsis*, *C.elegans*, and human as reference genome did not result in sufficiently high numbers of paralogs for meaningful statistical analysis.

A simple E-value cutoff proved to be as good as or better than the methods discussed above, hence we decided to use it as our primary method of paralog estimation.

2. The number of duplicates per gene and the distance to the nearest duplicate

Figure S2. Survival rates of single-gene knockouts as function of effective gene family size and sequence distance (E-value) to the closest paralog.

A. The effective gene family size is defined by BLAST E-value cutoff 10^{-10} as described in the main text (Methods). The effective gene family size *D* denotes the number of paralogs available for a given gene. Except for *E.coli* and worm, there are no or only weak correlations between *D* and the probability of survival *P(S)* of single-gene KOs.

B. The sequence distance is estimated as the E-value between the bait gene (targeted for single-KO) and its closest paralog. The histogram shows $–\log_{10}$(E-value) bins, with bin “0-5” denoting the least

closely, bin "30+" denoting the most closely related genes. For some organisms, i.e. *P. aeruginosis, E.coli*, yeast, and worm, there is moderate to good correlation between $-\log_{10}$(E-value) and the probability of survival *P(S)* of single-gene KOs.

Figures S3, S4, S5. The influence of ribosomal and WGD genes on single-KO survival rates in yeast.

As ribosomal genes and genes originating from the whole-genome duplication (WGD) have characteristics different to genes of other duplications, we compared survival rates of single- and double-gene knockouts (SKO, DKO) in the whole yeast genome (**Figure S3**) with those in the yeast genome without the WGD genes [4] (**Figure S4**) and those in the yeast genome without predicted ribosomal genes [5] (**Figure S5**). In each figure, the top (**A**) and middle panel **(B)** show the relationship between SKO survival rates and the E-value and effective gene family size, respectively. The bottom panel (**C**) shows the DKO survival rates in relationship to the effective gene family size. Note that an effective gene family size of *D=1* in SKO represents a two-gene family, for example, but in DKO it represents a three-gene family.

The enrichment of two-gene families in yeast in which both genes buffer for each other can in part be explained by the two-gene families originating from the WGD. However, when removing all WGD duplicates, trends are similar to those amongst all genes. Removal of ribosomal genes does not change any of the trends.

3. Expression analysis

To test for the influence of expression level on buffering by gene duplicates, we tested three different measures: i) experimental expression data; ii) Codon Bias Index (see main text); and iii) Codon Adaptation Index.

Experimental expression data

Figure S6. Chances of survival, fraction of genes with duplicates, and contribution of duplicates to survival in genes of different expression levels

Information on gene expression was collected for each organism from different sources (**Table S1**), filtering for experiments which used conditions (strain, medium) similar to those of the KO/KD screens. The figures shows survival *P(S),* the fraction of genes with duplicates *P(D≥1)* and the contribution of duplicates to survival *C* for the different subsets. * P-value < 0.01; ** P-value < 0.001

Figure S7. Survival P(S) and the effective gene family size D or the E-value between the target gene and its nearest duplicate at different expression levels (yeast)

At the example of yeast, we illustrate that within subsets of (highly) expressed genes the correlation between survival *P(S)* and the effective gene family size *D* (**A**) or the E-value between the target gene and its nearest duplicate (**B**) is similar to that of all genes. This trend is the same for *E.coli* and worm (*not shown*).

Codon Adaptation Index

Figure S8. Chances of survival, fraction of genes with duplicates, and contribution of duplicates to survival in genes of high or low CAI

To validate the findings from the experimental expression data, we used sequence-based approximations of expression levels, Codon Bias Index (CBI, main text) and Codon Adaptation Index (CAI). Both CBI and CAI were obtained from the CodonW server [6], using standard settings, but adjusting parameters for the respective organism. Calculation of CAI requires a reference data set of expressed genes for calculation of the index, whereas calculation of CBI is purely based on nucleotide

sequence. Both measures are expected to work less well in multi-cellular than in single-cellular organisms due to tissue-specific expression levels which cannot be captured by a single sequence feature.

We rank-ordered the values and selected subsets of genes with the highest or lowest CAI, respectively. The sizes of the subsets varied according to the organism's genome size. The figure shows survival *P(S)*, the fraction of genes with duplicates ($D\geq 1$), and the contribution of duplicates to buffering *C* in the top, middle and bottom panel, respectively. Both CBI and CAI show similar trends with respect to survival *P(S)*, the fraction of genes with duplicates ($D\geq 1$) and the buffering capacity *C* as the experimental expression data (main text and **Figure S7**).

4. Function analysis

Figure S9. Gene ontology annotation

Function annotation using the Gene Ontology vocabulary was downloaded from the GO website [7] and mapped to a generic GO Slim vocabulary from the same website. GO annotation was only available for yeast, worm, fly and mouse. The figures include functional categories with very few members (<30 genes) which have little statistical power.

A. The probability of survival *P(S)* for genes of different functions.

B. The contribution of duplicates to buffering *C* for genes of different functions.

Survival rates and buffering capacity vary widely across the different functional categories in the four organisms – function is not a correlate of buffering by duplicates. Similar to highly expressed genes, function categories of low *P(S)* tend to have higher contributions of duplicates to survival and *vice versa*. No bias for certain functions towards high or low buffering capacity occurs consistently across all organisms.

In yeast, duplicates do not buffer genes of growth and reproduction, but duplicates buffer kinases. In contrast, kinases and other cell cycle proteins in worm are hardly buffered by duplicates, while ribosomal proteins have a contribution of duplicates much higher than average. Due to multiple hypothesis testing, only P-values<0. 01 should be considered significant; none of the functional categories meet this threshold (Z-score across distribution of functional categories).

S – survival upon gene deletion; aa and derivative metab. – amino acid and derivatives metabolism; cellular component org. /biogen. – cellular component organization/biogenesis

Figure S10. Function annotation based on protein domains

Function annotations based on protein domains were obtained from the SUPERFAMILY database [5] using its domain predictions and the domain function annotation. The function annotation scheme consists of seven major categories which map to 50 smaller categories. The annotation was available for all organisms of our analysis. Probability of survival *P(S)* (top) and contribution of duplicates to buffering *C* (bottom) shown separately for seven organisms, shown for the major functional categories. Function categories of low *P(S)* tend to have higher contributions of duplicates to survival and *vice versa*.

5. Two-gene families as a model for buffering by duplicates

Previous studies reported that the contribution of duplicates to buffering when examined in double gene-KOs is very small (~2%)[1, 2]; however, this contribution is an underestimate for two reasons. First, those studies did not account for different gene family sizes. For example, if both genes of a two-gene family are knocked-out, the phenotype is more likely to be lethal than if two genes of a larger family are knocked-out and additional duplicates are available to buffer for loss of function. Second, two genes, which are unrelated by sequence similarity, can produce a viable double-KO phenotype i) because one or both genes are members of separate gene families in which the duplicates buffer for the KO; ii) because one or both genes are of functions that are not essential under tested conditions. This ambiguity with

double-KOs of unrelated genes inflates their chances of survival and makes them less valuable for analysis of buffering by duplicates.

Thus, in our analysis we explicitly distinguish between double-KOs of related and unrelated genes (**Figure S11**, middle/right). We also distinguish between different effective family sizes. We find that overall chances of survival of double-KOs are similar to that of single-KOs (*P(S)=0. 93 vs. P(S)=0. 82*) when focusing on double-KO targets which are sequence-related (**Figure S11**, middle) and excluding double-KOs of unrelated genes (**Figure S11**, right). In double-KOs of sequence-related genes (**Figure S11**, middle), overall survival rates are slightly elevated compared to those of single-KOs. This is most likely due to the *a priori* bias of double-KO experiments towards non-essentials genes - all genes tested by double-K0 screens where non-essential in single-KO screens.

We observe that the yeast genome is enriched for two-gene families whose members are likely to buffer for each other (**Figures S11B**). In single-KOs (**Figure S11B**, left), chances of survival are higher amongst two-gene families (*D=1*) than amongst singletons (*D=0*) and larger gene families (*D*≥*2*). In double-KOs of sequence-related genes (**Figure S11B**, middle), yeast two-gene families (*D=0*) have drastically reduced chances of survival. Two-gene families in yeast are enriched for duplicates originating from the whole-genome duplication (WGD)[4, 8], however, the observation from **Figure S11B** holds true even if members of the WGD are removed (**Figure S12**). Survival of all 609 two-gene families in our set (*P(S|D=1)=0. 51*; **Figure S11B** middle panel) is even lower than survival of the all WGD gene pairs (*P(S)=0. 86,* from ref. [8]). The enrichment of *buffering* two-gene families in yeast is also not due to preferential duplication of ribosomal genes (**Figure S12**), nor do we observe it in worm (**Figure S11C**).

Figure S11. Yeast two-gene families are enriched for buffering duplicates

Two-gene families play a special role in yeast. Survival upon single gene-KO is higher in two-gene than in larger families (left), i. e. the two-gene families are enriched for families in which both genes likely buffer for each other. If both genes of a two-gene family are knocked out, chances for survival are low (middle). The trends hold true even when accounting for whole-genome duplicates [8] or ribosomal genes (see **Figure S12**).

A. When examining survival upon single and double gene-KO in yeast, we distinguish between different buffering scenarios. The cartoons depict genes (circles) and their homologous relationships (lines) as predicted by sequence similarity. Filled circles (black) denote knocked-out genes. Left: single-KO of a singleton, a gene in a two- or three-gene family leaves zero, one or two additional duplicates, respectively, which can buffer for the KO. In the case of double gene knockouts, the two genes can either be sequence-related (i. e. homologous; middle) or unrelated (right). Middle: depending on the family size (two, three, four), after double-KO zero, one or more duplicates remain. Right: if the two double-KO genes are unrelated, zero (one, two) additional paralogs can be achieved if the genes are singletons (members of two-gene or three-gene families). In each group of buffering/KO scenarios (left, middle, right), the number of additional duplicates *D* is zero, one or more, while the actual family size varies.

B. Chances of survival upon gene-KO in yeast are comparable for single gene-KOs (left) and double gene-KO of sequence-related genes (middle). Survival is generally much higher in double gene-KOs of unrelated genes (right) since those two genes are unlikely to buffer for each other. Two-gene families in yeast are enriched for genes that buffer for each other: chances of survival are higher in single gene-KOs of two-gene families than of larger families. When both genes of a two-gene family are knocked-out, survival chances are low (middle). The red arrows point to the unusual behavior of yeast two-gene families. Numbers printed in the columns report the total number of tested genes (single gene-KO) or tested pairs (double gene-KO).

C. In worm, we observe trends similar to those in yeast (**B**). Duplicate genes increase chances of survival in single gene-KDs. For double gene-KDs, the situation is less clear, partly due to lower numbers of genes with KD information. In worm, there is no enrichment in buffering two-gene families as observed for yeast. Numbers printed in the columns report the total number of tested genes (single gene-KO) or tested pairs (double gene-KO).

Figure S12. Yeast two-gene families are enriched for buffering duplicates

Two-gene families play a special role in yeast, as explained in the main text. The families are enriched for duplicates which buffer for each other's loss of function, as can be seen in the comparatively high survival rates of two-gene families (*D=1*) in single-gene KOs and the low survival rates in of two-gene families (*D=0, related genes*) in double-KOs (both marked by arrows)(**A**). The trend holds true even if removing ribosomal genes (**B**) or genes of the whole-genome duplication (WGD) (**C**) [4]. Genes of the WGD are enriched for buffering two-gene families [8], but possibly not all WGD pairs have yet been identified. We also observe enrichment in buffering two-gene families in yeast in a set of predicted genetic interactions [2] (*not shown*).

Yeast

Two-gene families, when targeted for single- and double-gene KO, are a good set for studying characteristics of buffering by gene duplicates (see main text). Large- and small-scale double gene-KO tests identified 50 two-gene families with an SSL phenotype (buffering genes). The characteristics of these families are compared to characteristics of the 559 remaining two-gene families in yeast, and to the characteristics of nine two-gene families with viable phenotypes in double-KOs (**Table S3**).

Table S3B, D also describe the comparison of buffering and non-buffering two-gene families in terms of similarity of vectors describing their interactions. The vectors describe single gene-KO phenotypes [9], function [10], genetic interactions (from the large scale screens described in the main text as well as single SSL interactions listed in BioGRID [11]) and physical interactions (as listed in BioGRID [11]). The table lists several measures of vector similarity, of which the Jaccard index is used in the main text.

Duplicates from the WGD are known to have properties different to those of other duplicates [8, 12]. We tested some of the properties listed in **Table S3** for all 108 WGD two-gene families in comparison to the 501 two-gene families not identified to originate from the WGD [4] (**Table S4**). All tested properties are consistent with the findings on buffering in comparison to non-buffering genes. While there is a link between buffering capacity and origin of duplicates in the WGD (reflected in distinct protein characteristics), we cannot resolve causality. We hypothesize, however, that WGD gene pairs are strongly enriched in duplicates that buffer for each other's loss of function in single-gene deletions.

Worm

We extracted the 143 worm two-gene families tested in double-RNAi knockdowns by Tischler et al. [13] which resulted in 16 pairs of synthetic sick or lethal (SSL) phenotypes. We calculated the Codon Adaptation Index for the worm genes using a webserver, http://www.evolvingcode.net/codon/cai/cais.php [14], and found a significant bias similar to that in yeast (see main text), suggesting that buffering genes are more efficiently translated than non-buffering genes.

Tables

Table S1. Number of genes in subsets of expressed genes.

The table lists the number of genes in each subset as well as the number of essential genes in the subsets. Information on gene expression was collected for each organism from different sources (**Table S1**), filtering for experiments which used conditions (strain, medium) similar to those of the KO/KD screens.

For dual channel microarray experiments, we estimated expression levels based on the spot intensities in the microarrays. In all organisms (except for *M. genitalium* and mouse), we rank-ordered the quantitative expression levels and selected a subset of genes of the highest and lowest expression levels. Subsets were chosen proportional to dataset size. We tested different cutoffs for subset selection, all with the same qualitative results (*not shown*). In *M. genitalium* and mouse only protein identifications but no quantitative data was available, thus we divided the data into sets of 'expressed' (observed) and 'not expressed' (not observed) genes. The set of 'NOT Expressed Genes' is the set of all genes without the expressed genes. 'NOT Expressed Genes' can also be expressed, although at lower levels.

* - expression estimated from spot intensity; GEO – reference [15]; MGD – reference [16]; SMD – reference [17]

**Organism**	**Source of expression data**	**All Genes**	**Genes identified to be expressed or expressed at high levels**	**Genes identified to be not expressed or expressed at low levels**
H. pylorii	n/a	1559	n/a	n/a
M. genitatlium	Protein identification [18]	480	102	378
H. influenzae	GEO GSE5061	1704	600	600
M. tuberculosis	GEO GSE7588	3920	1200	1200
P aeruginosa	Avg. of four datasets GEO GSE2430, GSE3090, GSE4152, GSE5443	5566	1542	1440
B. subtilis	GEO GSM49830	4105	1430	368
E. coli	SMD dataset 15206	4234	1395	172
S. cerevisiae	Avg. of three datasets [19-21]	5318	2708	512
C. elegans	Gene expression at young adult * [22]	13891	170	1415
D. melanogaster	GEO GSM6159	12145	3635	250
M. musculus	From MGI TS26 Newborn Mouse	4267	2005	2262

Table S2. Sources of 14 yeast double-gene knockout screens

In addition to the main SGA screen by Tong et al. [1], several other large-scale studies have been conducted to-date. We compiled a list of 14 studies marked as 'systematic deletion screen' in GRID [11], which in total describe double-gene KOs of 204 baits against all non-essential yeast genes, resulting in 12,267 SSL interactions.

Number of SSL interactions in GRID	PubMed ID	Authors
3873	14764870	Tong AH et al. [1]
1010	16487579	Pan X et al. [23]
673	14690608	Krogan NJ et al. [24]
338	11743205	Tong AH et al. [25]
306	15715908	Lesage G et al. [26]
272	15766533	Zhao R et al. [27]
214	16157669	Daniel JA et al. [28]
191	15166135	Lesage G et al. [29]
180	15525520	Pan X et al. [30]
127	15725626	Loeillet S et al. [31]
94	16394103	Friesen H et al. [32]
94	15657441	Ingvarsdottir K et al. [33]
62	15817685	Menon BB et al. [34]
60	15238513	Suter B et al. [35]

Table S3. Properties of buffering and non-buffering yeast two-gene families

Yeast two-gene families, when targeted for single- and double-gene KO, are a good set for studying characteristics of buffering by gene duplicates. Large-scale and individual double gene-KO experiments have identified 50 two-gene families with an SSL phenotype (buffering pairs). However, only eight two-gene families tested in double-KOs have been found to have viable phenotypes (non-buffering pairs). Hence we also conducted all tests with an extended dataset of all two-gene families in yeast minus the 50 SSL two-gene families, resulting in 559 pairs.

The table lists all properties that we have tested for these sets of two-gene families. All properties were examined either *across* all genes in the respective set (**A, C**), or *between* the genes (**B, D**). Features from the calculations *across* genes are calculated *between* genes as *|feature(gene1)-feature(gene2)|*. Other features, e.g. sequence similarity, only exist *between* genes. Due to multiple hypothesis testing (~50 tests), a t-score>3.26 should be considered significant at an adjusted P-value of 0.05. Table **E** lists the numbers of orthologs and their essentiality (if known) for buffering and non-buffering gene pairs. Orthology is determined by InParanoid [3].

avg. – average

A. 50 buffering pairs vs. 559 two-gene families - Across genes

Source	Feature	Avg. 50 SSL genes	Count – 50 SSL	Avg – 559 background genes	Count – 559 background genes	t-score
Protein and mRNA expression [36]	Protein/mRNA	7142.09	11	7078.15	105	0.02
	mRNA abundance	4.95	91	4.96	928	-0.01
	Protein abundance	35040.36	29	56040.43	194	-1.40
SGD [37]	Molecular weight	1023.90	53	1742.84	459	-2.14
	PIso	7.37	99	7.64	1054	-1.20
	CAI	0.23	99	0.22	1054	0.59
	Length	588.26	99	525.54	1054	1.36
	CBI	0.19	99	0.18	1048	0.36
	FOP	0.52	99	0.51	1054	0.47
	GRAVY	-0.43	99	-0.40	1054	-0.89
	Aromaticity	0.09	99	0.09	1054	-0.67
	PEST_absolute counts	154.67	99	137.19	1054	1.38
	PEST_frequency	0.26	99	0.25	1054	1.23
Protein interactions [38]	No. protein-protein interactions	15.15	84	13.03	818	0.87
	PPixn_MIPS	23.33	36	31.58	214	-1.61
Functional network [10]	Clustering coefficient	0.29	96	0.32	875	-1.08
	Degree	27.64	98	20.69	946	2.32
InParanoid [3]	No. orthologs in 14 organisms	8.06	94	6.65	1001	2.34
Sequence features [39]	dN	0.120	56	0.170	552	**-4.43**
	dS (ajdusted)	2.11	56	2.16	552	-1.54
	dN/dS	0.06	56	0.08	552	**-4.39**
Protein production [40]	Protein production rate	0.63	90	0.55	920	0.50
	Proteins produced per mRNA	5.73	85	5.06	863	0.74
	Deletion grwoth rate	-0.17	87	-0.14	889	-0.75

	Transcription rate	0.11	85	0.10	863	0.46
	mRNA abundance	3.98	90	3.40	920	0.66
	mRNA decay rate	0.061	85	0.059	867	0.61
	Transcription rate / rel. translation rate	0.36	85	0.31	863	0.96
[41]	Protein half-life	108.5	74	127.7	704	-0.76

B. 50 buffering pairs vs. 559 two-gene families - Between genes

**Source**	**Feature**	**Avg. 50 SSL genes**	**Count – 50 SSL**	**Avg – 559 background genes**	**Count – 559 background genes**	**t-score**
Protein and mRNA expression [36]	Protein per mRNA	13224.000	2	7258.875	8	0.52
	mRNA abundance	2.555	43	4.045	431	-1.41
	Protein abundance	17394.283	7	42128.491	25	-1.21
SGD [37]	Molecular weight	723.833	18	2137.909	143	-1.91
	PIso	14938.606	33	22291.899	284	-1.15
	PIso	1.064	50	1.231	545	-0.98
	CAI	0.047	50	0.064	545	-1.76
	length	86.280	50	135.552	545	-2.28
	CBI	0.077	50	0.111	539	-2.80
	FOP	0.043	50	0.065	545	-2.93
	GRAVY	0.099	50	0.146	545	-2.99
	Arom	0.011	50	0.012	545	-0.63
	PEST_abs	33.140	50	43.431	545	-1.36
	PEST_rel	0.021	50	0.024	545	-1.03
Protein interactions [38]	No. protein interactions	5.351	37	6.045	332	-0.58
	No. protein interactions (MIPS)	0.941	17	1.358	81	-0.54
Functional network [10]	Clustering coefficient	0.162	48	0.202	406	-1.48
	Degree	14.980	49	14.077	457	0.39
InParanoid [3]	No. orthologs in 14 organisms	1.956	45	2.659	496	-1.01
Sequence features [39]	dN	0.038	17	0.086	176	**-4.64**
	dS	0.207	17	0.211	176	-0.10
	dN/dS	0.017	17	0.039	176	**-5.00**
Protein production [40]	Protein production rate	0.304	41	0.447	416	-1.36
	Proteins produced per mRNA	4.190	36	3.674	369	0.58
	Deletion grwoth rate	0.217	39	0.172	391	0.77
	Transcription rate	0.039	36	0.073	369	-2.98
	mRNA abundance	1.427	41	2.170	416	-1.74
	mRNA decay rate	0.030	36	0.026	373	0.98
	Transcription rate / rel. translation rate	8.039	36	8.252	373	-0.14
	Protein production rate	0.215	36	0.288	369	-1.19
[41]	Protein half-life	91.4	31	174.0	260	-1.93

Characteristics that only exist between genes.

**Source**	**Feature**	**Avg. 50 SSL**	**Count – 50**	**Avg – 559 backgroun**	**Count – 559**	**t-score**

		genes	SSL	d genes	background genes	
Functional network [10]	Shortest path	1.271	48	1.898	498	**-5.66**
BLAST output	Sequence similarity	54.332	50	46.795	555	2.15
Vector comparison: Similarity measure	Type of interaction					
Mutual information						
(data see paper)	Genetic	0.00	26	0.00	183	-0.28
[10]	Functional	0.01	23	0.00	394	0.79
[9]	Phenotype	0.02	10	0.03	177	-0.77
[11]	Physical	0.00	25	0.00	517	1.30
Jaccard index						
(data see paper)	Genetic	0.01	26	0.03	183	-0.63
[10]	Functional	0.15	23	0.11	394	1.11
[9]	Phenotype	0.17	10	0.23	177	-0.50
[11]	Physical	0.13	25	0.08	517	1.75
Avg. no. interactions per vector						
(data see paper)	Genetic	156.46	26	235.26	183	-0.75
[10]	Functional	4678.00	23	4678.00	394	1.00
[9]	Phenotype	100.00	10	100.00	177	1.00
[11]	Physical	5318.00	25	5318.00	517	1.00
Hamming distance						
(data see paper)	Genetic	6.27	26	6.42	183	-0.07
[10]	Functional	27.65	23	24.11	394	0.62
[9]	Phenotype	3.20	10	3.96	177	-0.86
[11]	Physical	24.24	25	19.09	517	1.06

C. 50 buffering pairs vs. 8 non-buffering pairs - Across genes

Source	Feature	Avg. 50 SSL genes	Count – 50 SSL	Avg – 8 non-SSL genes	Count – 8 non-SSL genes	t-score
Protein and mRNA expression [36]	Protein/mRNA	7142.091	11	3722.000	1	1.24
	mRNA abundance	4.948	91	0.906	14	**4.04**
	Protein abundance	35040.358	29	2115.875	4	2.84
SGD [37]	Molecular weight	66299.869	99	91885.000	16	-2.33
	PIso	7.367	99	7.576	16	-0.38
	CAI	0.232	99	0.134	16	**4.97**
	Length	588.263	99	821.625	16	-2.23
	CBI	0.187	99	0.051	16	**5.18**
	FOP	0.519	99	0.438	16	**5.36**
	GRAVY	-0.430	99	-0.523	16	1.36
	Aromaticity	0.086	99	0.086	16	-0.01
	PEST_absolute counts	154.667	99	258.688	16	-1.99
	PEST_frequency	0.257	99	0.293	16	-1.74
Protein interactions [38]	No. protein-protein interactions	15.155	84	4.286	14	**4.50**
Functional network [10]	Clustering coefficient	0.292	96	0.191	16	2.77
	Degree	27.643	98	21.063	16	1.54

InParanoid [3]	No. orthologs in 14 organisms	8.064	94	5.800	15	1.52
Sequence features [39]	dN	0.120	56	0.240	8	-1.80
	dS (ajdusted)	2.112	56	2.111	8	0.01
	dN/dS	0.056	56	0.113	8	-1.95
Protein production [40]	Protein production rate	0.632	90	0.056	12	**3.45**
	Proteins produced per mRNA	5.733	85	1.388	11	**4.07**
	Deletion grwoth rate	-0.168	87	-0.058	12	-2.48
	Transcription rate	0.109	85	0.040	11	2.87
	mRNA abundance	3.976	90	1.250	12	3.18
	mRNA decay rate	15.675	85	13.119	11	1.12
	Transcription rate / rel. translation rate	0.359	85	0.595	11	-1.67
[41]	Protein half-life	108.5	74	177.1	13	-0.50

D. 50 buffering pairs vs. 8 non-buffering pairs - Between genes

Source	Feature	Avg. 50 SSL genes	Count – 50 SSL	Avg – 8 non-SSL genes	Count – 8 non-SSL genes	t-score
Protein and mRNA expression [36]	mRNA abundance	2.555	43	0.430	7	2.43
	Protein abundance	17394.283	7	4727.490	1	1.35
SGD [37]	Molecular weight	9552.460	50	34705.000	8	**-3.45**
	PIso	1.064	50	0.854	8	1.07
	CAI	0.047	50	0.014	8	**3.57**
	Length	86.280	50	305.500	8	**-3.45**
	CBI	0.077	50	0.061	8	0.94
	FOP	0.043	50	0.033	8	0.93
	GRAVY	0.099	50	0.144	8	-1.39
	Aromaticity	0.011	50	0.006	8	1.61
	PEST_absolute counts	33.140	50	93.625	8	-2.65
	PEST_frequency	0.021	50	0.020	8	0.14
Protein interactions [38]	No. protein-protein interactions	5.351	37	3.500	6	1.10
Functional network [10]	Clustering coefficient	0.162	48	0.133	8	0.52
	Degree	14.980	49	11.625	8	0.51
InParanoid [3]	No. orthologs in 14 organisms	1.956	45	2.286	7	-0.26
Sequence features [39]	dN	0.038	17	0.006	1	0.00
	dS (ajdusted)	0.207	17	0.245	1	0.00
	dN/dS	0.017	17	0.012	1	0.00
Protein production [40]	Protein production rate	0.304	41	0.082	5	2.14
	Proteins produced per mRNA	4.190	36	1.910	4	1.52
	Deletion growth rate	0.217	39	0.071	5	2.15
	Transcription rate	0.039	36	0.019	4	2.25
	mRNA abundance	1.427	41	0.840	5	1.41
	mRNA decay rate	0.030	36	0.020	4	1.32
	Transcription rate / rel. translation rate	0.215	36	0.516	4	-1.83
[41]	Protein half-life	91.4	31	407.47	5	-0.90

Characteristics that only exist between genes.

Source	Feature	Avg. 50 SSL genes	Count – 50 SSL	Avg – 8 non-SSL genes	Count – 8 non-SSL genes	t-score
Functional network [10]	Shortest path	1.271	48	1.625	8	-1.26
BLAST output	Sequence similarity	54.332	50	32.486	8	**4.91**
Vector comparison: Similarity measure	Type of interaction					
Mutual information						
[11]	Physical	0.005	25	0.001	8	2.03
(data see paper)	Genetic	0.003	26	0.028	7	-1.72
[10]	Functional	0.006	23	0.001	7	2.03
[9]	Phenotype	0.022	10	0.010	2	0.45
Jaccard index						
[11]	Physical	0.13	25	0.03	8	2.01
(data see paper)	Genetic	0.01	26	0.07	7	-1.49
[10]	Functional	0.15	23	0.04	7	2.04
[9]	Phenotype	0.17	10	0.11	2	0.27
Avg. no. interactions per vector						
[11]	Physical	5318.00	25	5318.00	8	1.00
(data see paper)	Genetic	156.46	26	1389.86	7	**-3.23**
[10]	Functional	4678.00	23	4678.00	7	1.00
[9]	Phenotype	100.00	10	100.00	2	1.00
Hamming distance						
[11]	Physical	24.24	25	9.75	8	1.92
(data see paper)	Genetic	6.27	26	23.00	7	**-3.16**
[10]	Functional	27.65	23	11.57	7	1.88
[9]	Phenotype	3.20	10	8.50	2	**-3.71**

E. 50 buffering pairs vs. 559 two-gene families – Orthologs

	Buffering pairs	Non-buffering pairs
**Single ortholog in fly, worm or mouse**		
- essential	11	55
- non-essential	13	116
**Multiple orthologs in fly, worm or mouse (inparalogs)**		
- all essential	1	2
- all non-essential	6	42
**Other (mix of the above)**	24	148

Table S4. Properties of yeast WGD two-gene families and non-WGD two-gene families

Duplicates arising from the whole genome duplication (WGD) are different to other duplicates [8]. WGD genes are also enriched in pairs of SSL interaction [8], thus likely to buffer for each other. Conversely, some of the properties of the 'buffering' genes discussed in our paper (see main text and **Table S2**) may be accounted to the enrichment of WGD genes amongst buffering genes, although the enrichment of WGD in buffering genes is not significant (**Table S2**). We tested some of the properties listed in **Table S2** for all WGD two-gene families in comparison to two-gene families not identified to originate from the WGD. All properties are consistent with the findings on buffering in comparison to non-buffering gene.

	**Two-gene families from WGD**	**Two-gene families not from WGD**	**df**	**t-score**	**P-value**
**N=**	501	108			
**Protein degradation (protein half life)** [41]	104	124.4	561	0.97	0.335
**Protein abundance – APEX** [36]	122259.3	107691.1	103	-0.45	0.653
**Average protein abundance (Western, 2D, APEX)** [36]	27778.3	25229.0	645	-0.41	0.682
**Average mRNA abundance (SAGE, genomic, HDA)** [36]	6.3	4.3	820	-1.95	0.052
**Protein/mRNA ratio** [36]	3570.8	10483.4	640	1.08	0.280
**CAI (Codon Adaptation Index)** [37]	0.3	0.2	853	-2.36	0.019

References

1. Tong AH, Lesage G, Bader GD, Ding H, Xu H, Xin X, Young J, Berriz GF, Brost RL, Chang M *et al*: Global mapping of the yeast genetic interaction network. *Science* 2004, 303(5659):808-813.
2. Wong SL, Zhang LV, Tong AH, Li Z, Goldberg DS, King OD, Lesage G, Vidal M, Andrews B, Bussey H *et al*: Combining biological networks to predict genetic interactions. *Proc Natl Acad Sci U S A* 2004, 101(44):15682-15687.
3. Remm M, Storm CE, Sonnhammer EL: Automatic clustering of orthologs and in-paralogs from pairwise species comparisons. *J Mol Biol* 2001, 314(5):1041-1052.
4. Kellis M, Birren BW, Lander ES: Proof and evolutionary analysis of ancient genome duplication in the yeast Saccharomyces cerevisiae. *Nature* 2004, 428(6983):617-624.
5. Wilson D, Madera M, Vogel C, Chothia C, Gough J: The SUPERFAMILY database in 2007: families and functions. *Nucleic Acids Res* 2007, 35(Database issue):D308-313.
6. CodonW: http://bioweb.pasteur.fr/seqanal/interfaces/condonw.html.
7. Harris MA, Clark J, Ireland A, Lomax J, Ashburner M, Foulger R, Eilbeck K, Lewis S, Marshall B, Mungall C *et al*: The Gene Ontology (GO) database and informatics resource. *Nucleic Acids Res* 2004, 32(1):D258-261.
8. Guan Y, Dunham MJ, Troyanskaya OG: Functional Analysis of Gene Duplications in Saccharomyces cerevisiae. *Genetics* 2007, 175(2):933-943.
9. McGary KL, Lee I, Marcotte EM: Broad network-based predictability of Saccharomyces cerevisiae gene loss-of-function phenotypes. *Genome Biol* 2007, 8(12):R258.
10. Lee I, Date SV, Adai AT, Marcotte EM: A probabilistic functional network of yeast genes is accurate, extensive, and highly modular. *Science* 2004, 306(5701):1555-1558.
11. Stark C, Breitkreutz BJ, Reguly T, Boucher L, Breitkreutz A, Tyers M: BioGRID: a general repository for interaction datasets. *Nucleic Acids Res* 2006, 34(Database issue):D535-539.
12. Davis JC, Petrov DA: Do disparate mechanisms of duplication add similar genes to the genome? *Trends Genet* 2005, 21(10):548-551.
13. Tischler J, Lehner B, Chen N, Fraser AG: Combinatorial RNA interference in C. elegans reveals that redundancy between gene duplicates can be maintained for more than 80 million years of evolution. *Genome Biol* 2006, 7(8):R69.
14. Wu G, Culley DE, Zhang W: Predicted highly expressed genes in the genomes of Streptomyces coelicolor and Streptomyces avermitilis and the implications for their metabolism. *Microbiology* 2005, 151(Pt 7):2175-2187.
15. Barrett T, Troup DB, Wilhite SE, Ledoux P, Rudnev D, Evangelista C, Kim IF, Soboleva A, Tomashevsky M, Edgar R: NCBI GEO: mining tens of millions of expression profiles--database and tools update. *Nucleic Acids Res* 2006.
16. Eppig JT, Blake JA, Bult CJ, Kadin JA, Richardson JE: The mouse genome database (MGD): new features facilitating a model system. *Nucleic Acids Res* 2007, 35(Database issue):D630-637.
17. Gollub J, Ball CA, Sherlock G: The Stanford Microarray Database: a user's guide. *Methods Mol Biol* 2006, 338:191-208.
18. Wasinger VC, Pollack JD, Humphery-Smith I: The proteome of Mycoplasma genitalium. Chaps-soluble component. *Eur J Biochem* 2000, 267(6):1571-1582.
19. Velculescu VE, Zhang L, Zhou W, Vogelstein J, Basrai MA, Bassett DE, Jr., Hieter P, Vogelstein B, Kinzler KW: Characterization of the yeast transcriptome. *Cell* 1997, 88(2):243-251.
20. Holstege FC, Jennings EG, Wyrick JJ, Lee TI, Hengartner CJ, Green MR, Golub TR, Lander ES, Young RA: Dissecting the regulatory circuitry of a eukaryotic genome. *Cell* 1998, 95(5):717-728.
21. Wang Y, Liu CL, Storey JD, Tibshirani RJ, Herschlag D, Brown PO: Precision and functional specificity in mRNA decay. *Proc Natl Acad Sci U S A* 2002, 99(9):5860-5865.
22. Jiang M, Ryu J, Kiraly M, Duke K, Reinke V, Kim SK: Genome-wide analysis of developmental and sex-regulated gene expression profiles in Caenorhabditis elegans. *Proc Natl Acad Sci U S A* 2001, 98(1):218-223.
23. Pan X, P. Y, Yuan DS, Wang X, Bader JS, Boeke JD: A DNA integrity network in the yeast Saccharomyces cerevisiae. *Cell* 2006, 124(5):1069-1081.
24. Krogan NJ, Keogh MC, Datta N, Sawa C, Ryan OW, Ding H, Haw RA, Pootoolal J, Tong AH, Canadien V *et al*: A Snf2 family ATPase complex required for recruitment of the histone H2A variant Htz1. *Molecular Cell* 2003, 12(6):1565-1576.
25. Tong AH, Evangelista M, B. PA, Xu H, Bader GD, Page N, Robinson M, Raghibizadeh S, Hogue CW, Bussey

H *et al*: Systematic genetic analysis with ordered arrays of yeast deletion mutants. *Science* 2001, 294(5550):2364-2368.
26. Lesage G, Shapiro J, Specht CA, Sdicu AM, Menard P, Hussein S, Tong AH, Boone C, Bussey H: An interactional network of genes involved in chitin synthesis in Saccharomyces cerevisiae. *BMC Genet* 2005, 6(1):8.
27. Daniel JA, Keyes BE, Ng YP, Freeman CO, Burke DJ: Diverse functions of spindle assembly checkpoint genes in Saccharomyces cerevisiae. *Genetics* 2006, 172(1):53-65.
28. Lesage G, Sdicu AM, Menard P, Shapiro J, Hussein S, Bussey H: Analysis of beta-1,3-glucan assembly in Saccharomyces cerevisiae using a synthetic interaction network and altered sensitivity to caspofungin. *Genetics* 2004, 167(1):35-49.
29. Zhao R, Davey M, Hsu YC, Kaplanek P, Tong A, Parsons AB, Krogan N, Cagney G, Mai D, Greenblatt J *et al*: Navigating the chaperone network: an integrative map of physical and genetic interactions mediated by the hsp90 chaperone. *Cell* 2005, 120(5):715-727.
30. Friesen H, Humphries C, Ho Y, Schub O, Colwill K, Andrews B: Characterization of the yeast amphiphysins Rvs161p and Rvs167p reveals roles for the Rvs heterodimer in vivo. *Mol Biol Cell* 2006, 17(3):1306-1321.
31. Loeillet S, Palancade B, Cartron M, Thierry A, Richard GF, Dujon B, Doye V, Nicolas A: Genetic network interactions among replication, repair and nuclear pore deficiencies in yeast. *DNA Repair (Amst)* 2005, 4(4):459-468.
32. Pan X, Yuan DS, Xiang D, Wang X, Sookhai-Mahadeo S, Bader JS, Hieter P, Spencer F, Boeke JD: A robust toolkit for functional profiling of the yeast genome. *Mol Cell* 2004, 16(3):487-496.
33. Ingvarsdottir K, Krogan NJ, Emre NC, Wyce A, Thompson NJ, Emili A, Hughes TR, Greenblatt JF, Berger SL: H2B ubiquitin protease Ubp8 and Sgf11 constitute a discrete functional module within the Saccharomyces cerevisiae SAGA complex. *Mol Cell Biol* 2005, 25(3):1162-1172.
34. Menon BB, Sarma NJ, Pasula S, Deminoff SJ, Willis KA, Barbara KE, Andrews B, Santangelo GM: Reverse recruitment: the Nup84 nuclear pore subcomplex mediates Rap1/Gcr1/Gcr2 transcriptional activation. *Proc Natl Acad Sci U S A* 2005, 102(16):5749-5754.
35. Suter B, Tong A, Chang M, Yu L, Brown GW, Boone C, Rine J: The origin recognition complex links replication, sister chromatid cohesion and transcriptional silencing in Saccharomyces cerevisiae. *Genetics* 2004, 167(2):579-591.
36. Lu P, Vogel C, Wang R, Yao X, Marcotte EM: Absolute protein expression profiling estimates the relative contributions of transcriptional and translational regulation. *Nat Biotechnol* 2007, 25(1):117-124.
37. Nash R, Weng S, Hitz B, Balakrishnan R, Christie KR, Costanzo MC, Dwight SS, Engel SR, Fisk DG, Hirschman JE *et al*: Expanded protein information at SGD: new pages and proteome browser. *Nucleic Acids Res* 2007, 35(Database issue):D468-471.
38. Gavin AC, Bosche M, Krause R, Grandi P, Marzioch M, Bauer A, Schultz J, Rick JM, Michon AM, Cruciat CM *et al*: Functional organization of the yeast proteome by systematic analysis of protein complexes. *Nature* 2002, 415(6868):141-147.
39. Wall DP, Hirsh AE, Fraser HB, Kumm J, Giaever G, Eisen MB, Feldman MW: Functional genomic analysis of the rates of protein evolution. *Proc Natl Acad Sci U S A* 2005, 102(15):5483-5488.
40. Fraser HB, Hirsh AE, Giaever G, Kumm J, Eisen MB: Noise minimization in eukaryotic gene expression. *PLoS Biol* 2004, 2(6):e137.
41. Belle A, Tanay A, Bitincka L, Shamir R, O'Shea EK: Quantification of protein half-lives in the budding yeast proteome. *Proc Natl Acad Sci U S A* 2006, 103(35):13004-13009.

A.

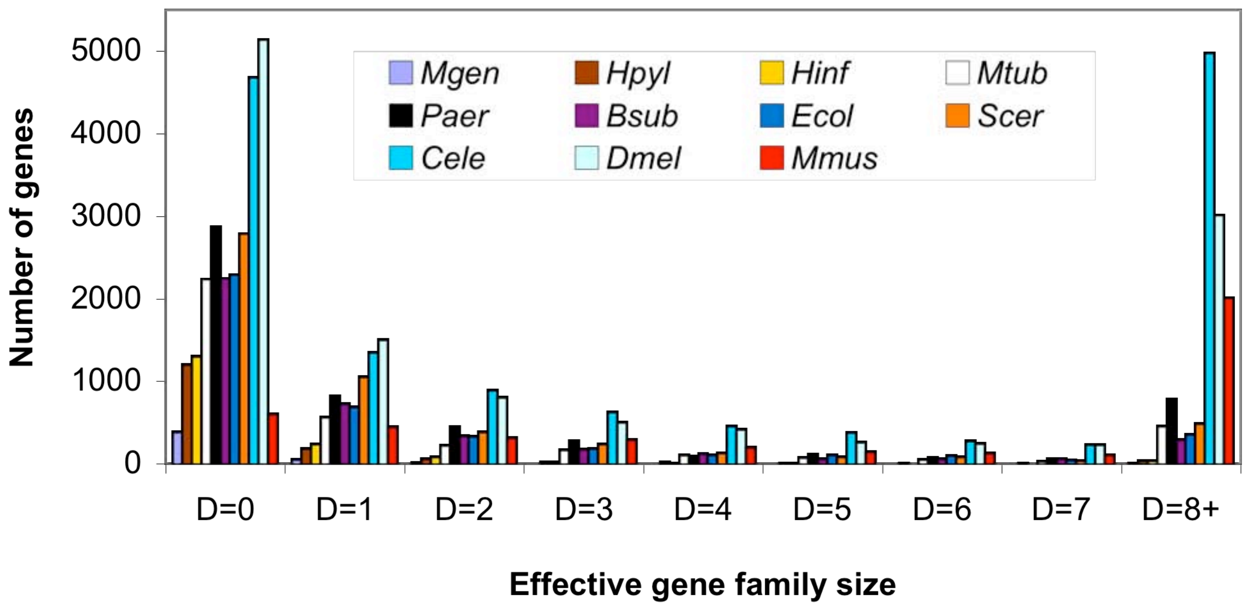

B.

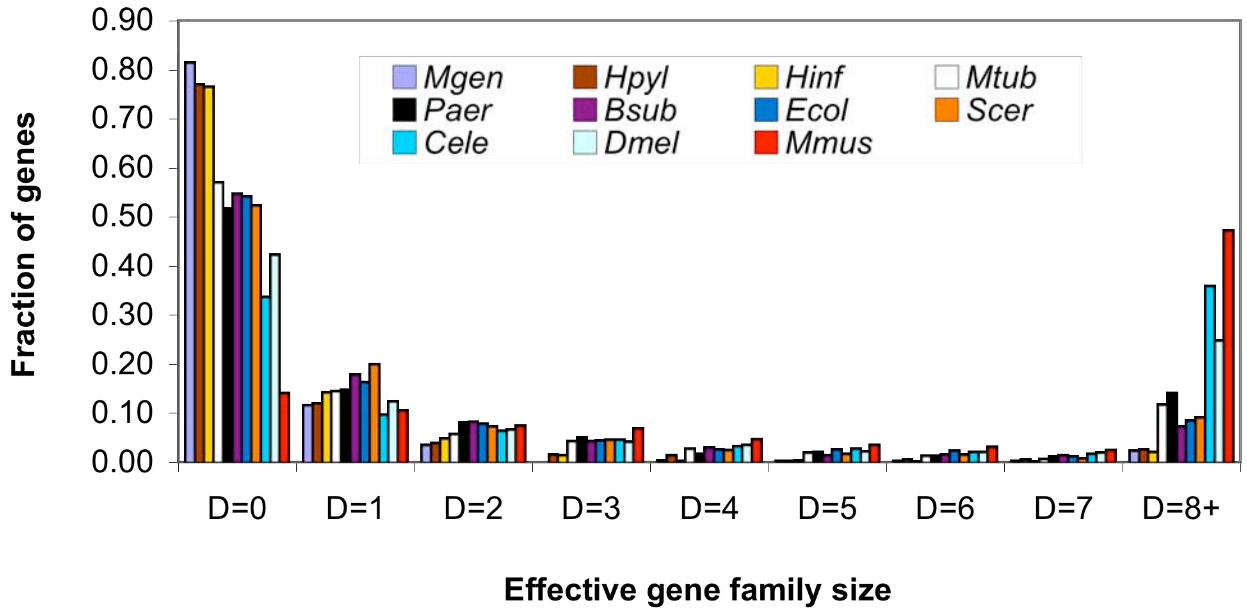

Figure S1. Gene family size distribution for all genomes in our analysis

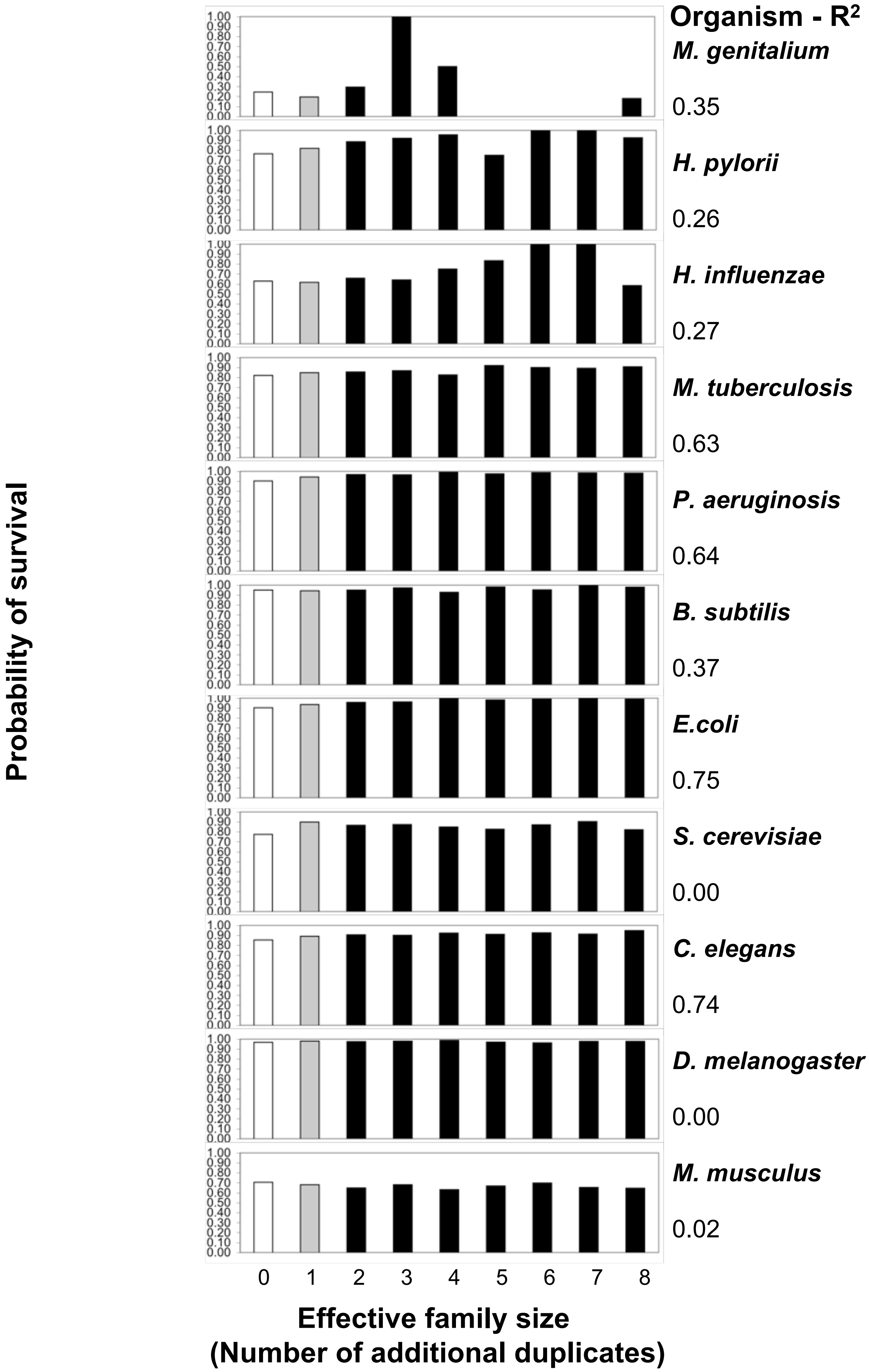

Figure S2A. Survival rates of single-gene knockouts as function of effective gene family size and sequence distance (E-value) to the closest paralog

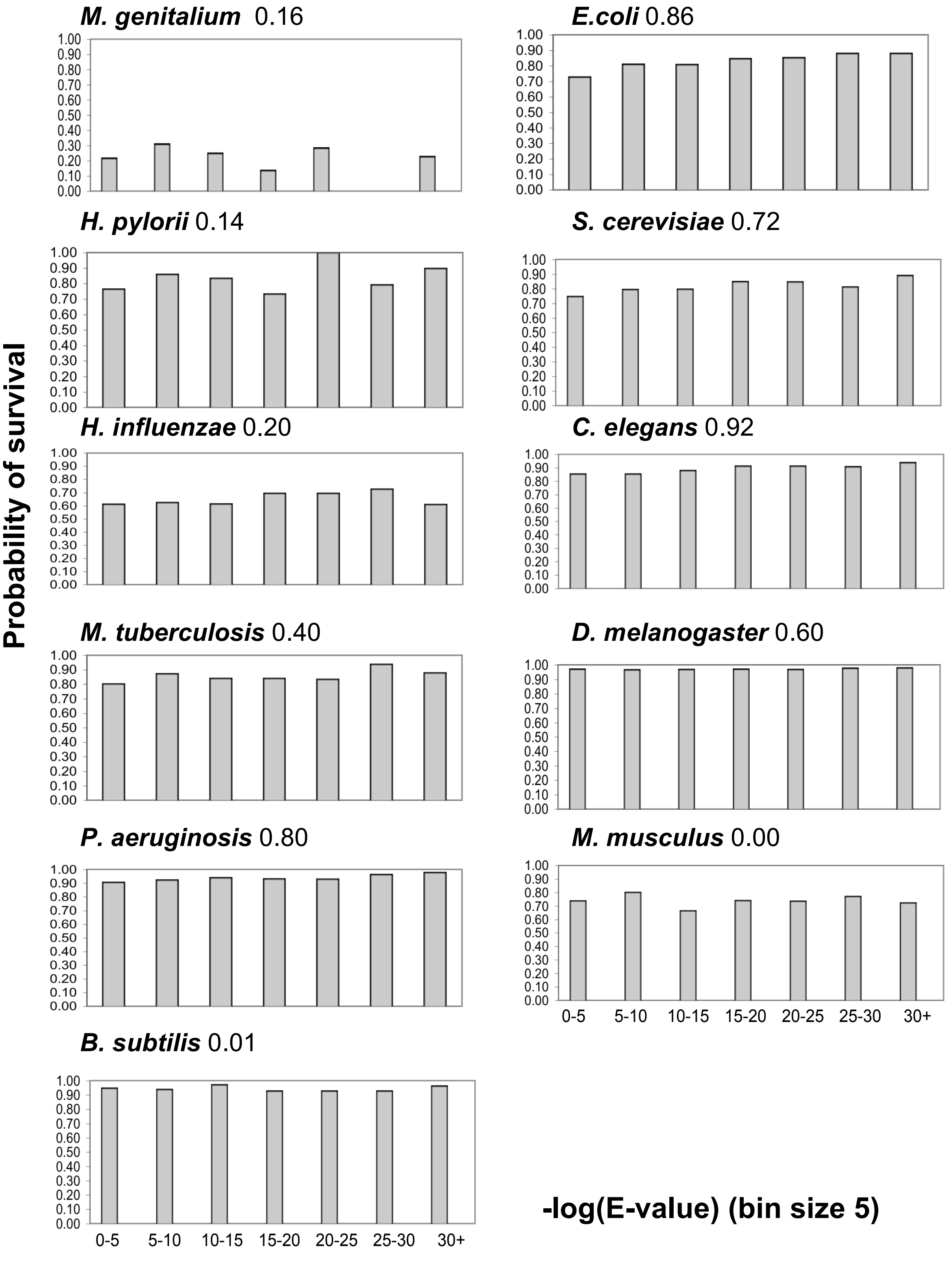

Figure S2B. Survival rates of single-gene knockouts as function of effective gene family size and sequence distance (E-value) to the closest paralog

A. Yeast SKO (regular data) R2=0.72

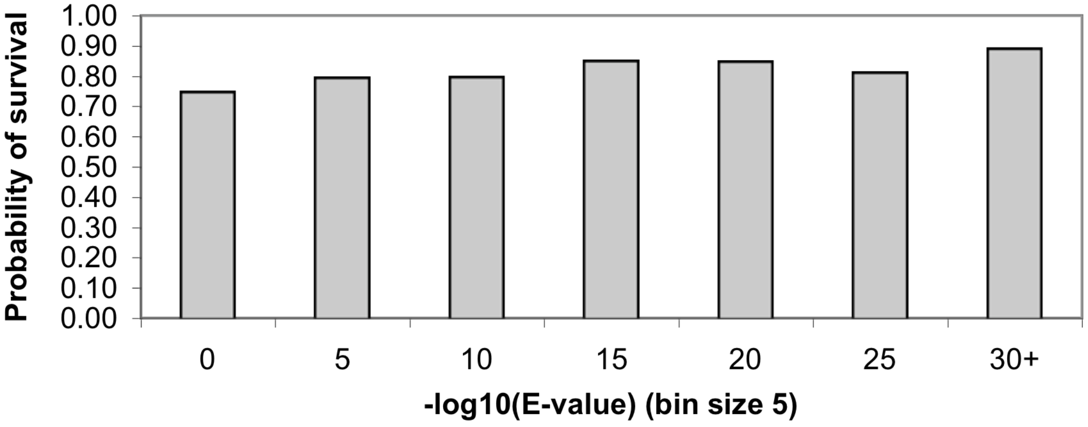

B. Yeast SKO (regular data) R2=0.00

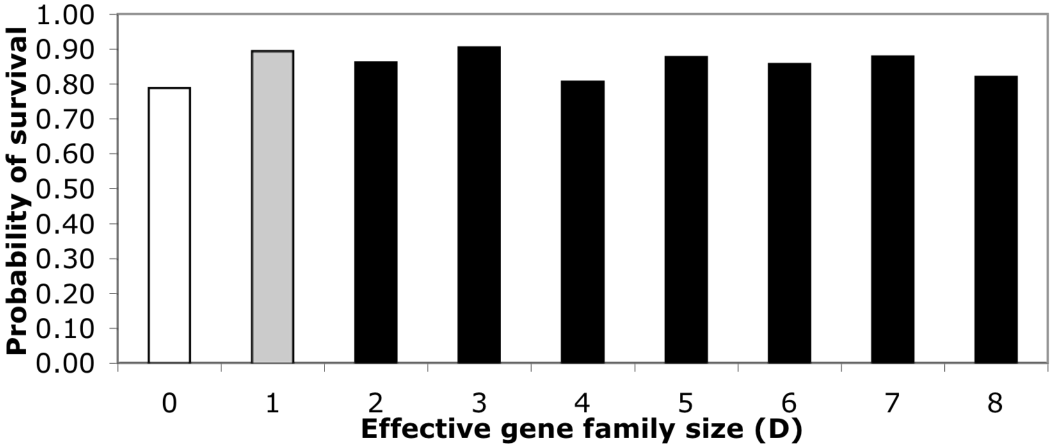

C. Yeast DKO (regular data) R2=0.16

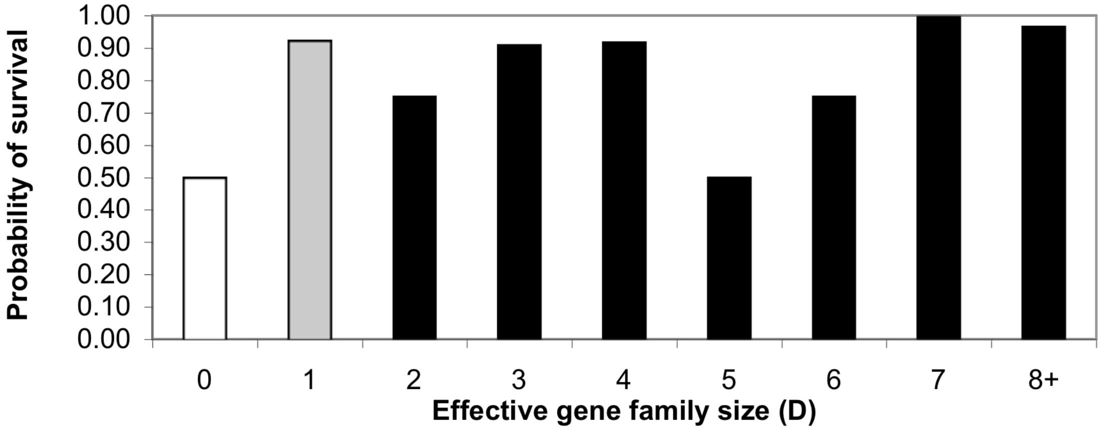

Figures S3. The influence of ribosomal and WGD genes on single-KO survival rates in yeast

A. Yeast SKO (minus WGD) R2=0.32

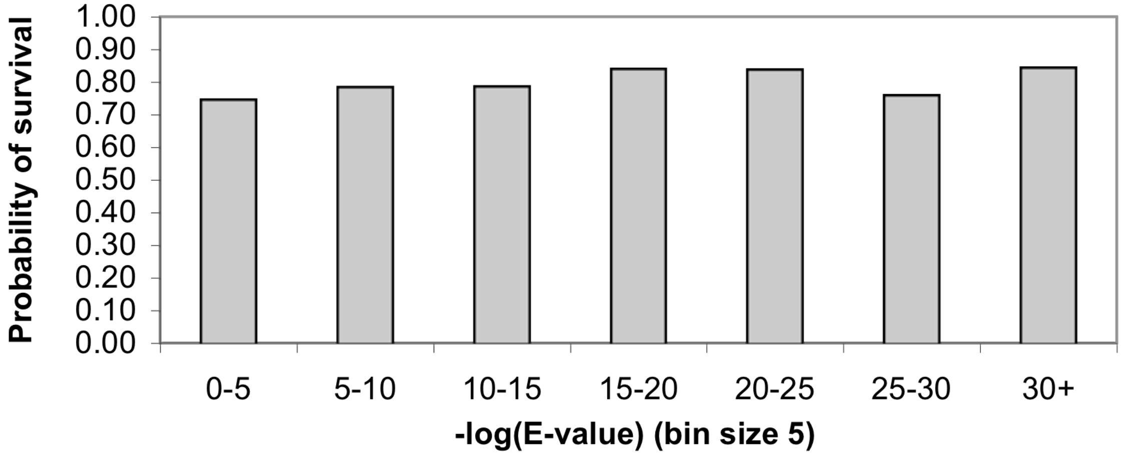

B. Yeast SKO (minus WGD) R2=0.00

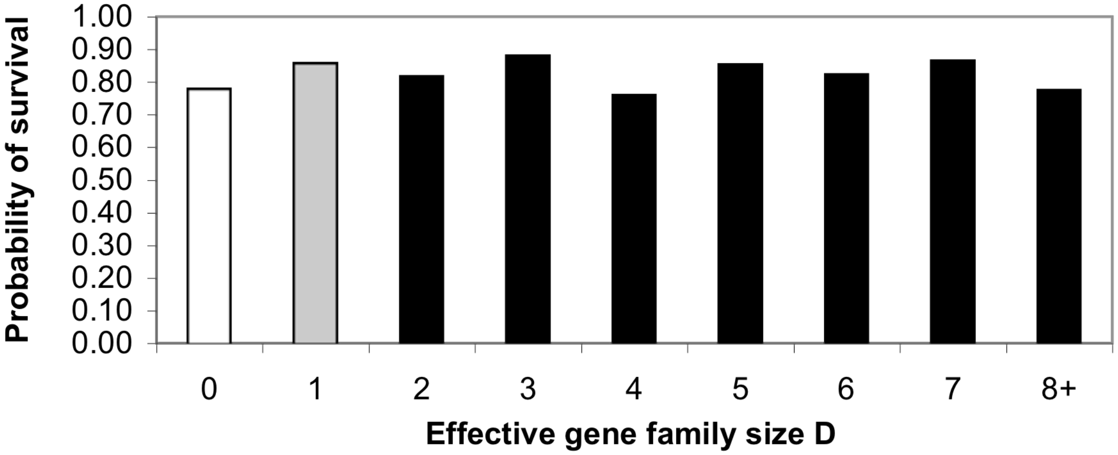

C. Yeast DKO (minus WGD) R2=0.12

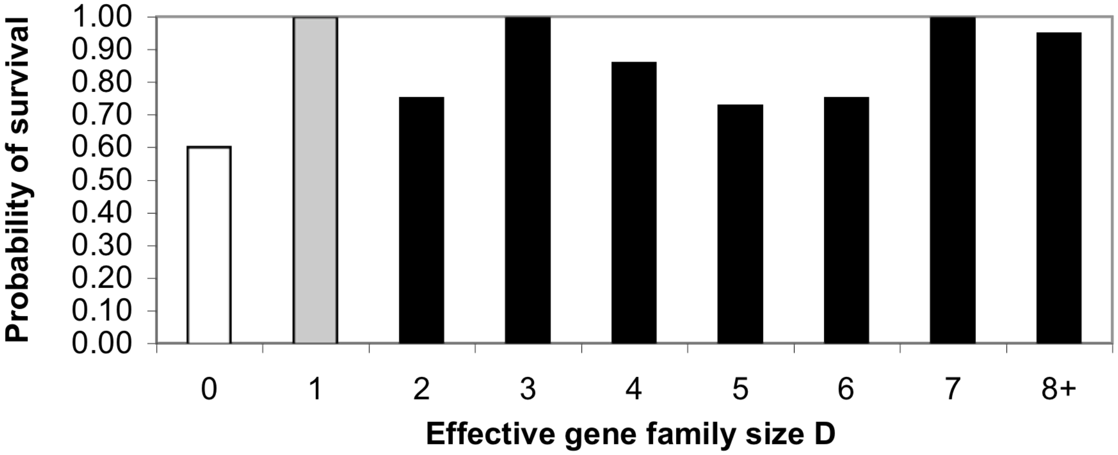

Figures S4. The influence of ribosomal and WGD genes on single-KO survival rates in yeast

A. Yeast SKO (minus ribosomal genes) R2=0.77

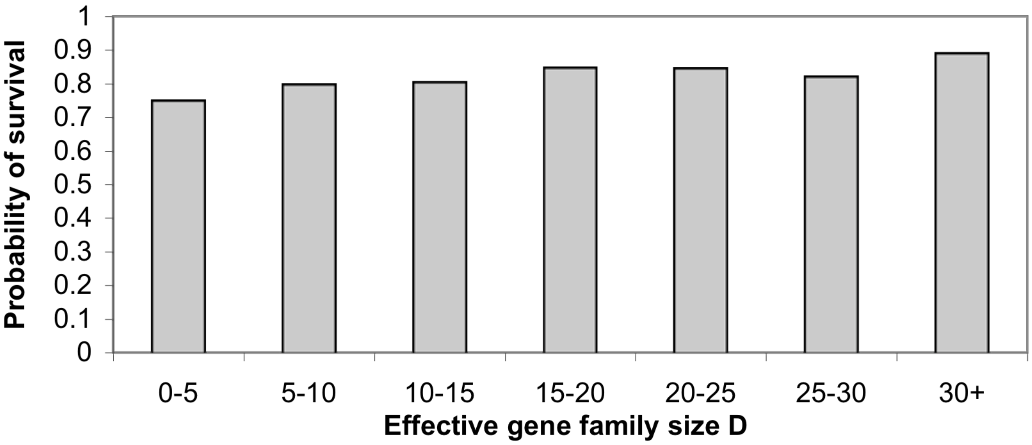

B. Yeast SKO (minus ribosomal genes) R2=0.00

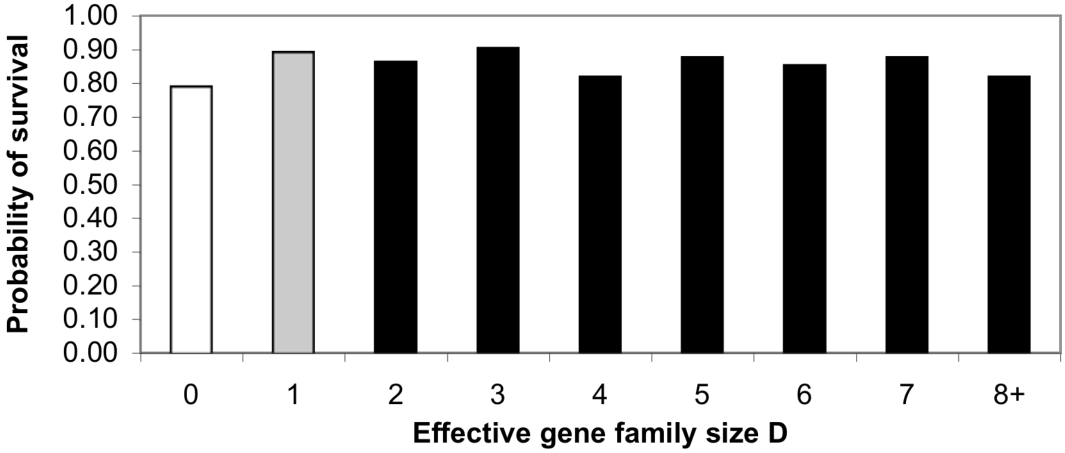

C. Yeast DKO (minus ribosomal genes) R2=0.16

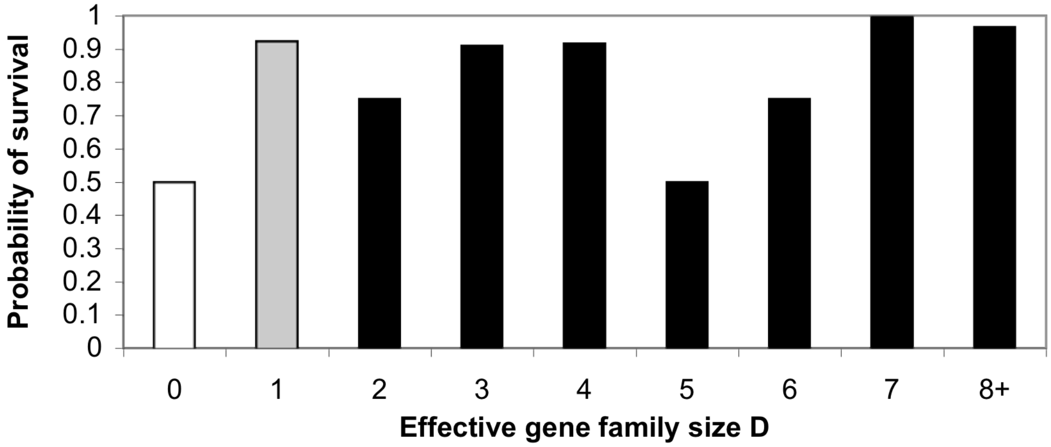

Figures S5. The influence of ribosomal and WGD genes on single-KO survival rates in yeast

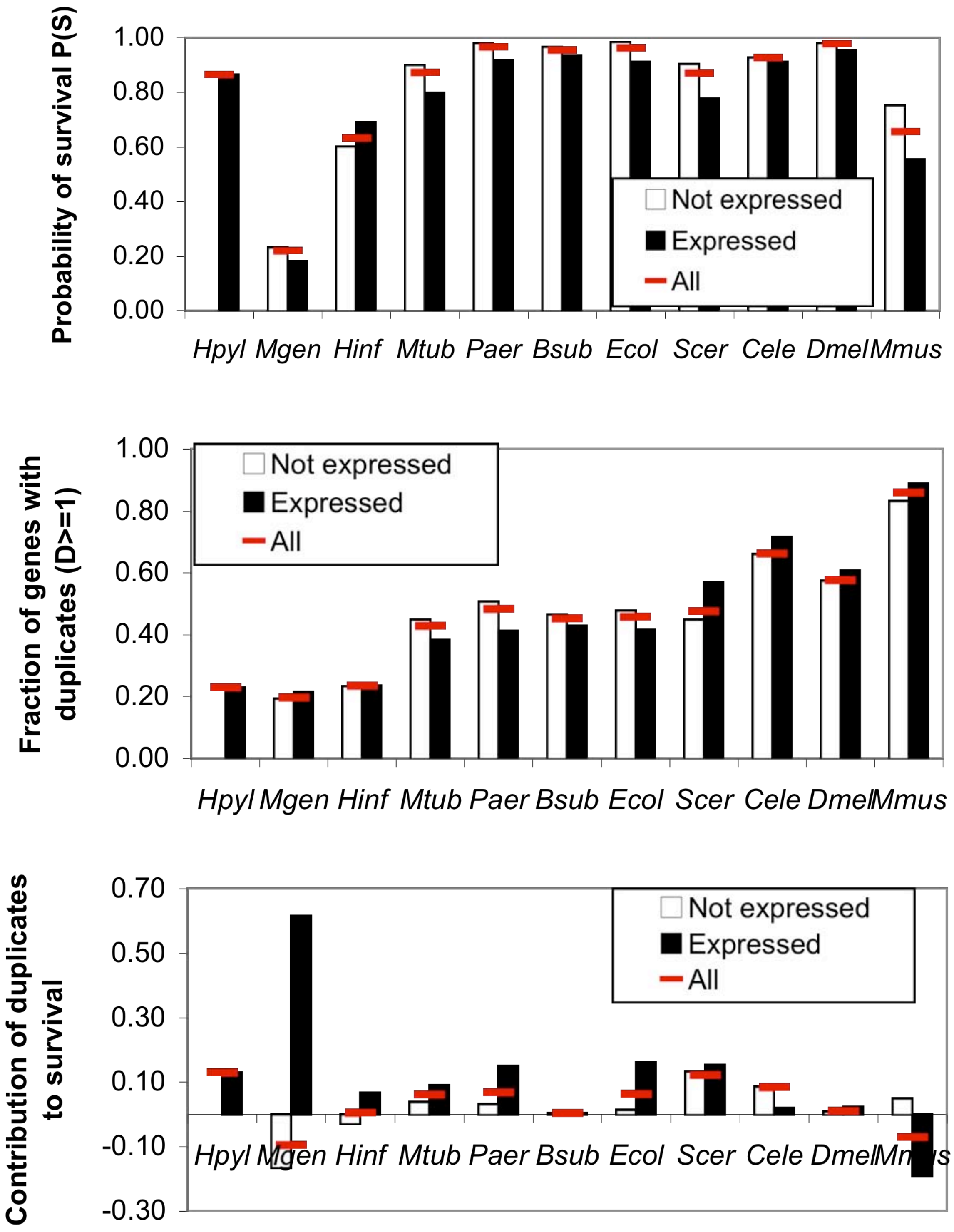

Figure S6. P(S), P(D>=1) and C in genes of different expression levels (experimental data). Expression data for H. pylori is missing.

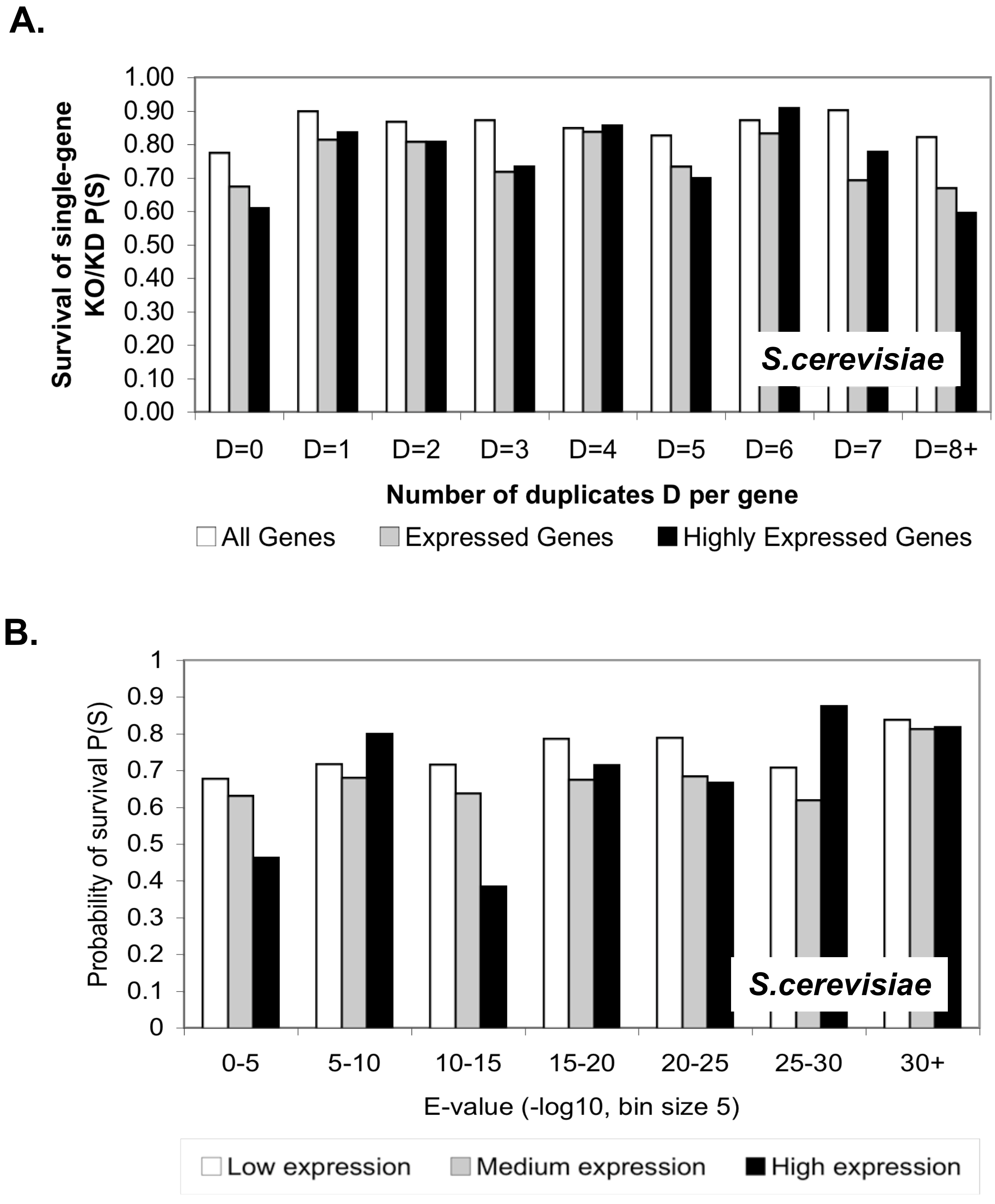

Figure S7. Survival P(S) and the effective gene family size D or the E-value between the target gene and its nearest duplicate at different expression levels (experimental data)

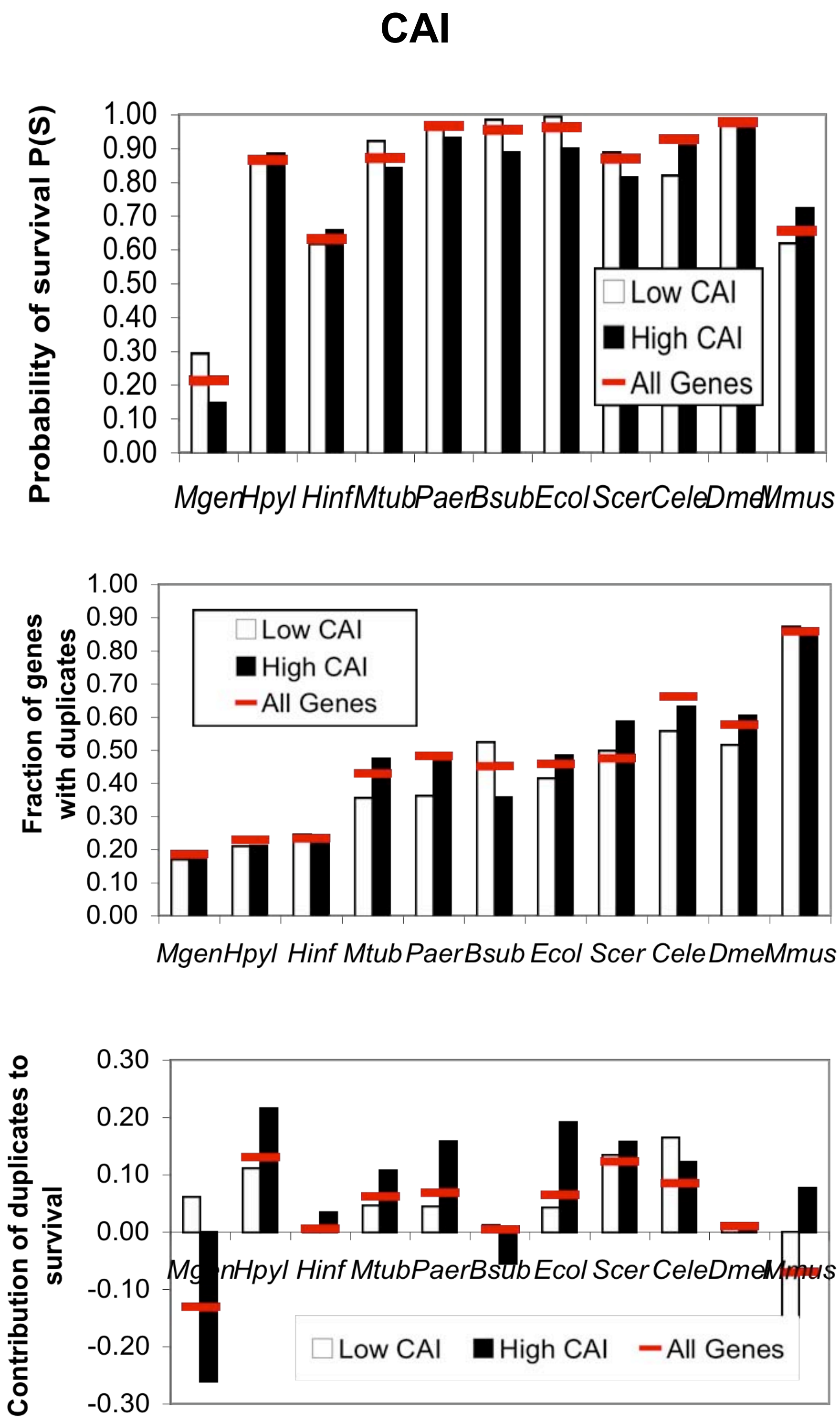

Figure S8. P(S), P(D>=1) and C in genes of high or low Codon Bias Index/Codon Adaptation Index (CBI/CAI)

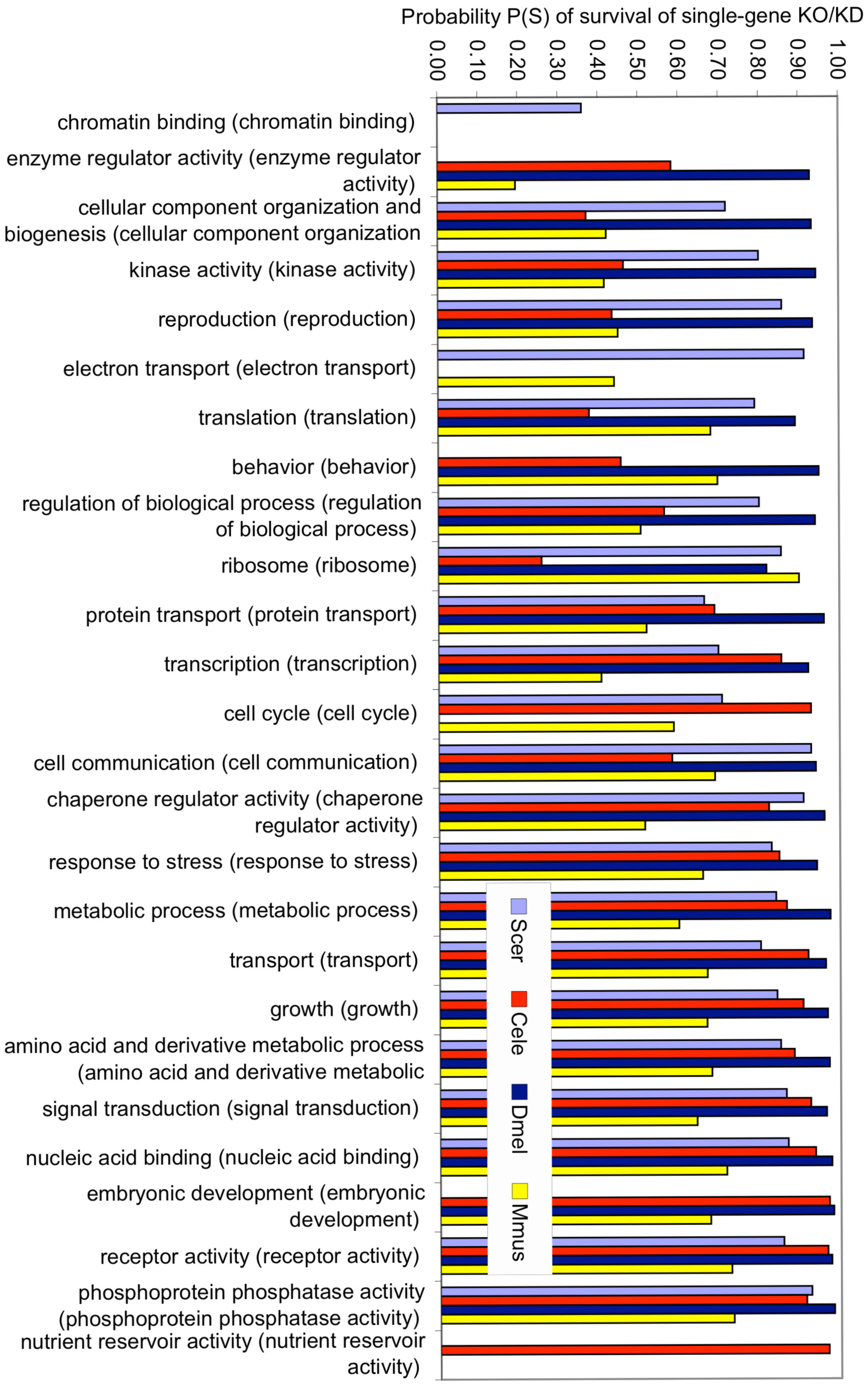

Figure S9A. Function annotation -- Gene Ontology

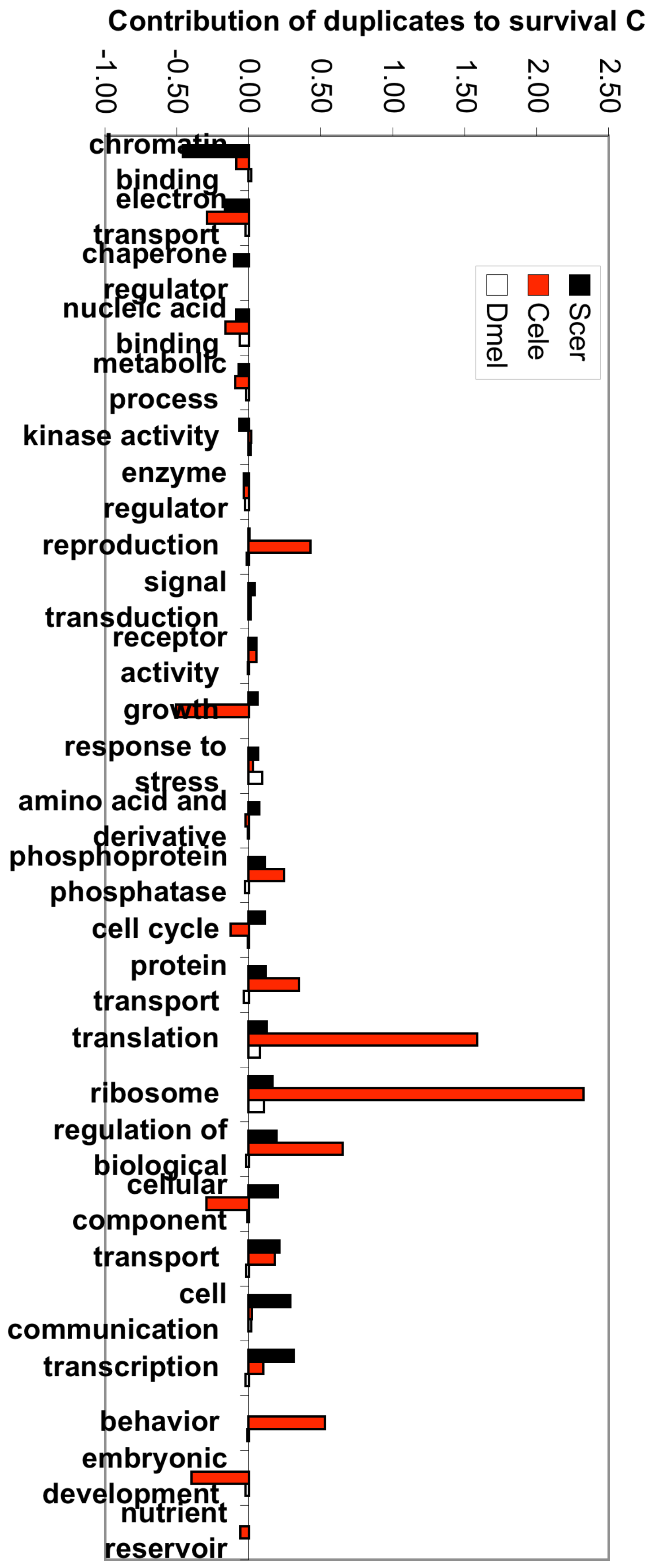

Figure S9B. Function annotation -- Gene Ontology

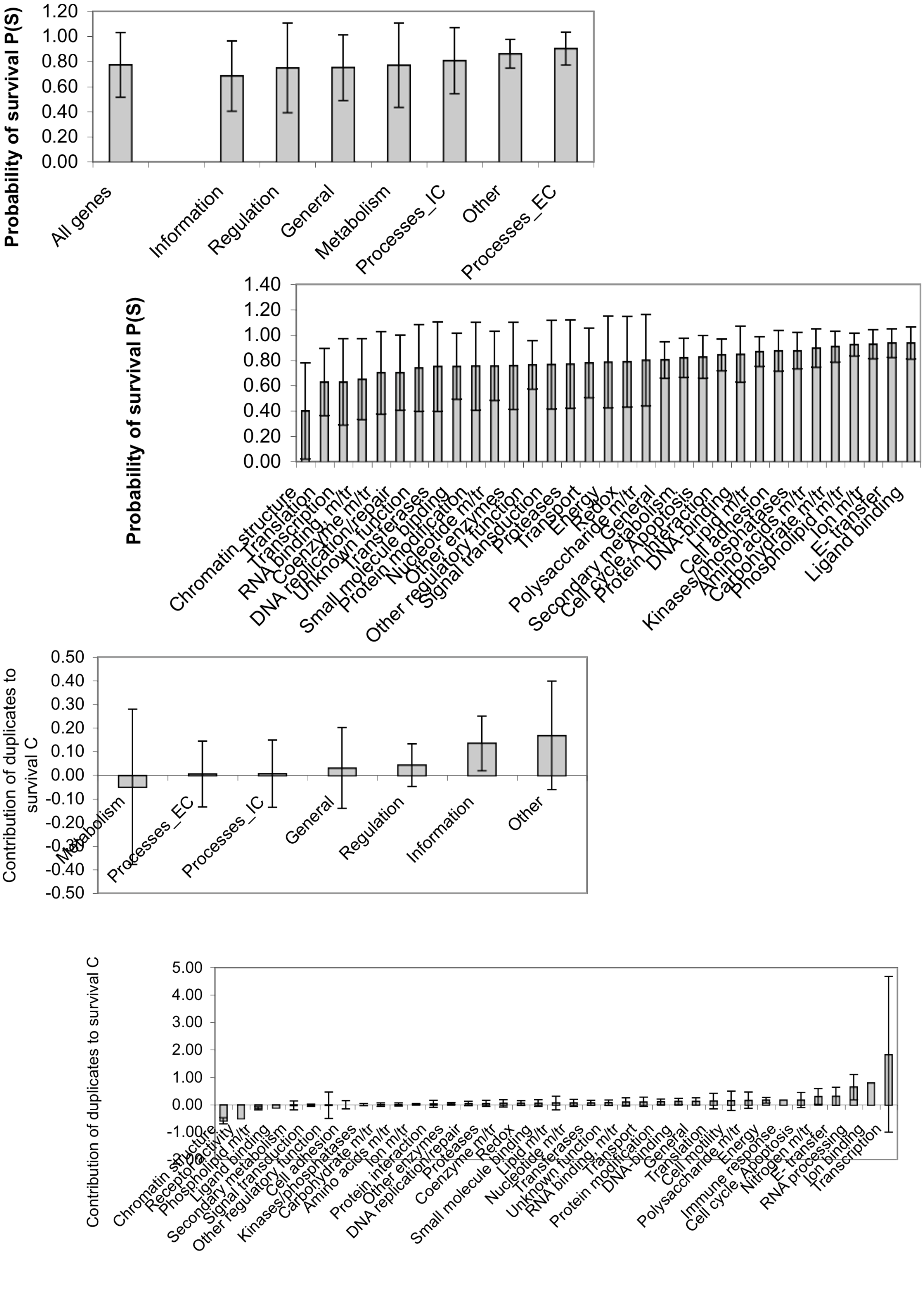

Figure S10. Function annotation based on protein domains

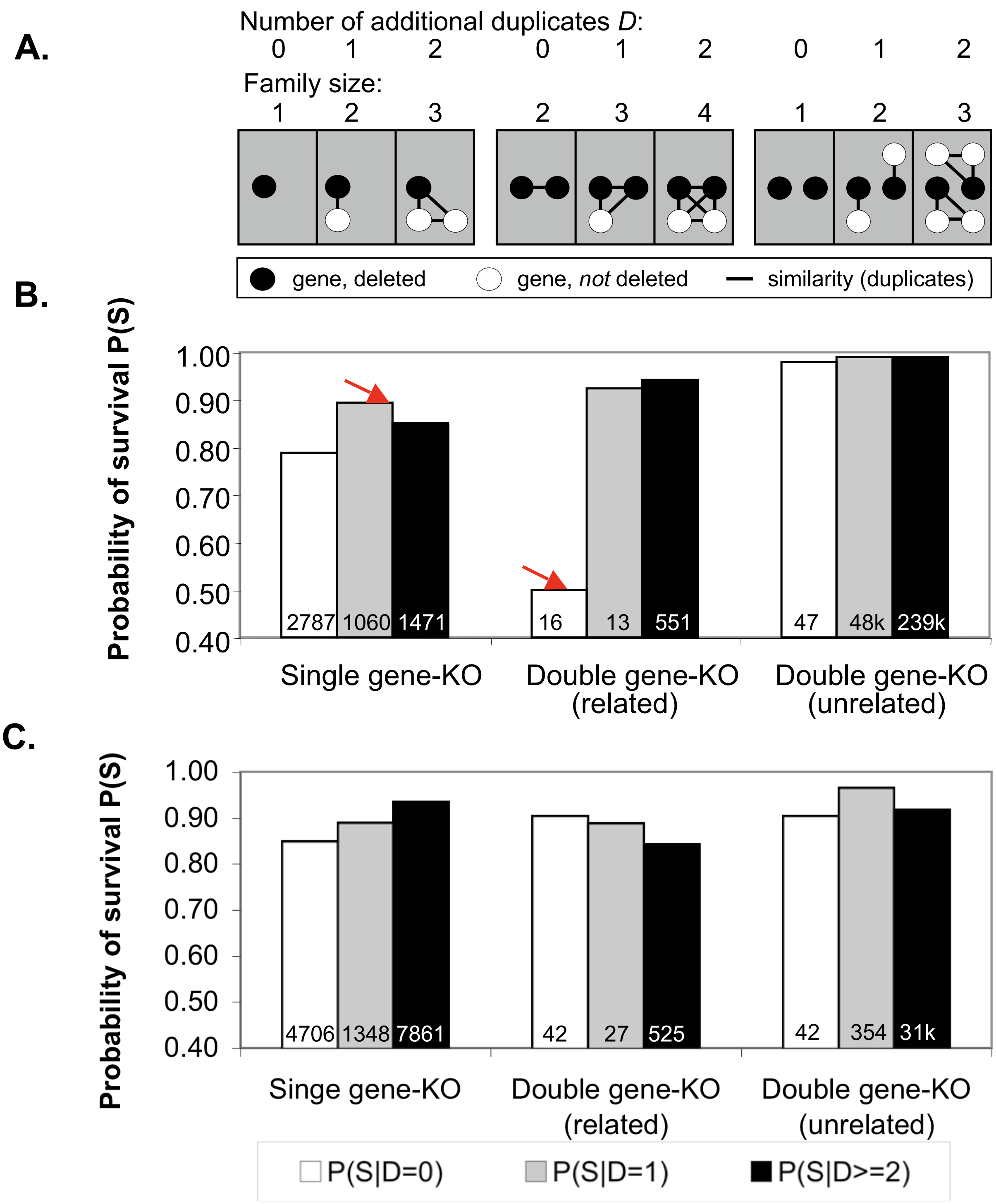

Figure S11. Yeast two-gene families are enriched for buffering duplicates

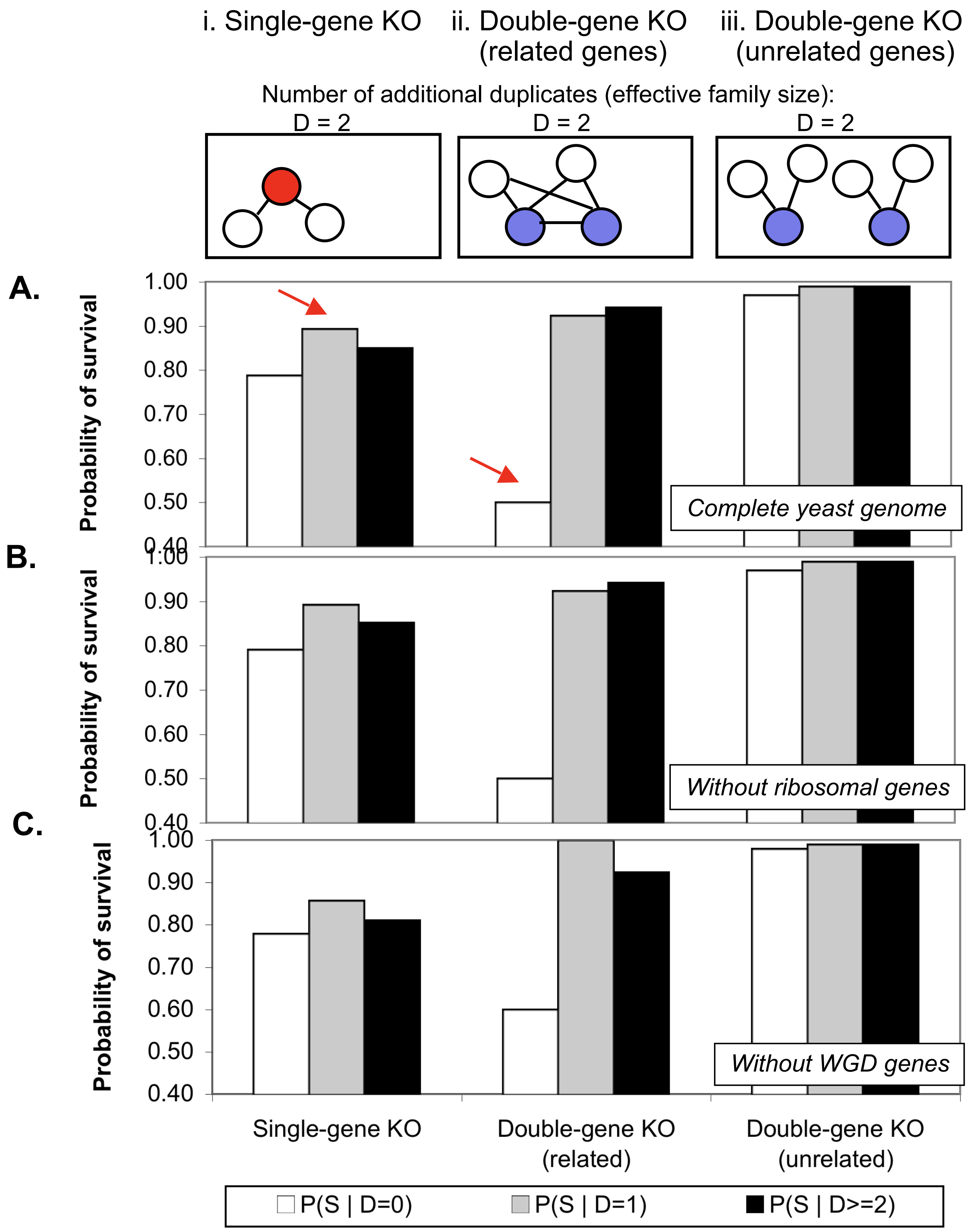

Figure S12. Yeast two-gene families are enriched for buffering duplicates
